# Improvement of symptoms in children with autism by TOMATIS training: a cross-sectional and longitudinal study

**DOI:** 10.3389/fnbeh.2024.1357453

**Published:** 2024-03-18

**Authors:** Yujia Fu, Mei Tian, Jiaxi Chen, Wenfeng Chen, Huang Li

**Affiliations:** ^1^Department of Psychology, Renmin University of China, Beijing, China; ^2^Hai Perui Education Technology Co., Ltd, Shanghai, China; ^3^Affiliated Haixia Hospital of Huaqiao University, Quanzhou, China

**Keywords:** autism spectrum disorder, TOMATIS, training, music intervention, behavior improvement

## Abstract

**Introduction:**

Autism spectrum disorder (ASD) is a neurological condition that is marked by deficits in social interaction, difficulty expressing oneself, lack of enthusiasm, and stereotypical conduct. The TOMATIS training method is an effective music therapy for children with ASD for its individually developed programs to improve behavioral deficits.

**Methods:**

The research employed both longitudinal and crosssectional designs.

**Results:**

In the cross-sectional study, the experimental group showed significant improvement in symptoms after TOMATIS training compared to the control group of children with ASD. The results validated the effect of TOMATIS treatment for ASD-related deficits, including perceptual-motor, attentional, social, and emotional issues.

**Discussion:**

ASD’s auditory hypersensitivity hampers social information processing, but TOMATIS enhances cochlear frequency selectivity, aiding in capturing relevant auditory stimuli. In addition, the longitudinal study confirmed these findings, which proved TOMATIS training effective in clinically treating ASD. This study focused on audiometric indicators and behavioural improvement, elucidating the mechanisms behind the training’s success. Behavioral improvements might stem from TOMATIS’ frequency selectivity, reshaping auditory organ-cortical feedback loops to filter interference and focus on valid information.

## 1 Introduction

The presence of Autism Spectrum Disorder (ASD) can lead to social interaction issues and repetitive stereotyping, resulting in serious difficulties for individuals in their professional, personal, and academic lives (American Psychiatric Association and Association, 1994). Children with ASD often struggle with eye-to-eye gaze, gross motor, fine motor, language, attention, and learning ability, accompanied by auditory processing issues, particularly in verbal processing and social communication, which remain incurable (Corbett et al., 2008). People with ASD exhibit atypical reactions to auditory stimuli in contrast to individuals with normal development. The auditory pathway exhibits a wide range of diverse manifestations in this atypical response, including heightened processing of both localized and simple stimuli, as well as reduced processing of holistic and complex stimuli (O’Connor, 2012; Ouimet et al., 2012).

Although the pathogenesis of ASD has not yet been determined, many treatments have been developed to alleviate the various symptoms of ASD. Cognitive behavioral therapy (CBT) is known to be the most effective intervention available for treating ASD symptoms. Effective treatment requires gradual risk-taking and conscious behavior modification through exposure or behavioral experimentation (Hollocks et al., 2023). It is not clear which cognitive characteristics may influence treatment response. In addition to this, autism spectrum disorders (ASD) are heterogeneous and may have different etiologies and different phenotypes (Cho et al., 2023). Therefore, interventions that explore individual specificity and effective behavior change are necessary. For children with ASD, a potentially narrow perceptual time window may have cascading effects on higher-order cognitive functions (Stevenson et al., 2014; Kawakami et al., 2020). Individuals with ASD spectrum disorders often experience sensory processing issues due to the improper processing of vestibular, proprioceptive, and tactile sensations in the brain. It is noteworthy, however, that people with ASD are more adept at recognizing the pitch of isolated pure tone stimuli than those without the condition when it comes to pitch discrimination and categorization tasks (Ouimet et al., 2012; Rabeyron et al., 2020). Whereas musical interventions act on the basal senses, this offers some possible advantages.

Both Auditory Integration Therapy (AIT) and music therapy can be used as alternative therapies in previous studies. Zhang et al. (2018) reviewed the current common methods of ASD music therapy interventions in the world, which mainly include schools of thought such as Nordoff-Robbins music therapy or creative music therapy, psychodynamic music therapy, music therapy applying behavioral modification techniques, somatic music therapy, auditory integrative therapy, and neurological music therapy, and the TOMATIS method is similar to AIT but uses frequency slicing. And there are few reports focusing on this method. In previous studies, a number of effective music therapies exist, such as improvisational music therapy (Geretsegger et al., 2016), music therapy group interventions (LaGasse, 2014), and family-centered music therapy (Thompson et al., 2014), among others. The French physician Alfred A. Tomatis (1920–2001) made the discovery of the TOMATIS EFFECT (TE) in the 1950s, as he held the belief that the sense of hearing, mental attitudes, speech, and language were closely linked. The TOMATIS EFFECT served as the foundation for Alfred A. Tomatis’ invention of the Electronic Ear (EE) and the discovery of the TOMATIS EFFECT by Ross-Swain (2007), as it specifically targets the frequencies audible, and if a faulty ear is provided with the chance to perceive the accurate sound, sound processing is instantly and subconsciously enhanced. Ross-Swain (2007) discovered that TOMATIS auditory stimulation enhanced auditory processing abilities in children diagnosed with ASD. Additionally, TOMATIS demonstrated a positive impact on interpreting performance and sleep quality (du Toit et al., 2011). Some researchers assert that listening to various music styles diminishes galvanic skin responses and generates joyful and calming experiences (Alvarsson et al., 2010; Roque et al., 2013). The TOMATIS effect states that music can be customized to address an individual’s unique deficit areas in children with ASD. The TOMATIS effect is more effective than intensive behavioral training because it uses a lower student-teacher ratio, includes a home environment, and involves a minimum of 20–25 h of training per week, with ongoing evaluation and modification of intervention goals and objectives during the intervention (Brbić and Tomić, 2020). TOMATIS training allows for the enhancement of auditory integration and tolerance to sound by exercising the small muscles in the middle ear, the reticular system, and cortical connections. Therefore, this study sought to explore the effectiveness of TOMATIS as a possible intervention.

Previous studies have typically been exploratory or experimental (El-Tellawy et al., 2022), and over the past 10 years, there have been investigations conducted either through qualitative research methods such as interviews and narratives (Blum, 2015), or with populations like adults and other hearing-impaired individuals (Dursun et al., 2021), or through discrete experiments investigating the effects of language and social skills (Peters-Scheffer, 2008). As research into music therapy approaches continues, it has been established that music in specific frequency regions is closely linked to specific functional abilities (Vervoort et al., 2008). Sensory integration, encompassing balance, rhythm, coordination, muscle tone, body awareness, sense of direction, and laterality, is primarily associated with low-frequency sounds in the first zone (0–750H); speech, memory, attention, and voice control are primarily linked to mid-frequency sounds in the second zone (750–4,000 Hz); and energetic intuition, ideas, spiritual creativity, and auditory cohesion are primarily associated with high-frequency sounds in the third zone (4,000 Hz and above). Previous research has yet to elucidate the dissimilarities in the impacts and processes of these varying music frequencies to ameliorate the symptoms of children with ASD. In order to tackle these problems, the current research endeavors to implement a novel music therapy strategy for children with ASD in China, incorporating the TOMATIS technique, which has demonstrated efficacy in various domains, into the intervention for children with ASD. It was hypothesized that children who received TOMATIS training would show greater improvement in autism-related symptoms and perform better at long-term compared to untrained children.

## 2 Methods

### 2.1 Participants

**Figure 1:**
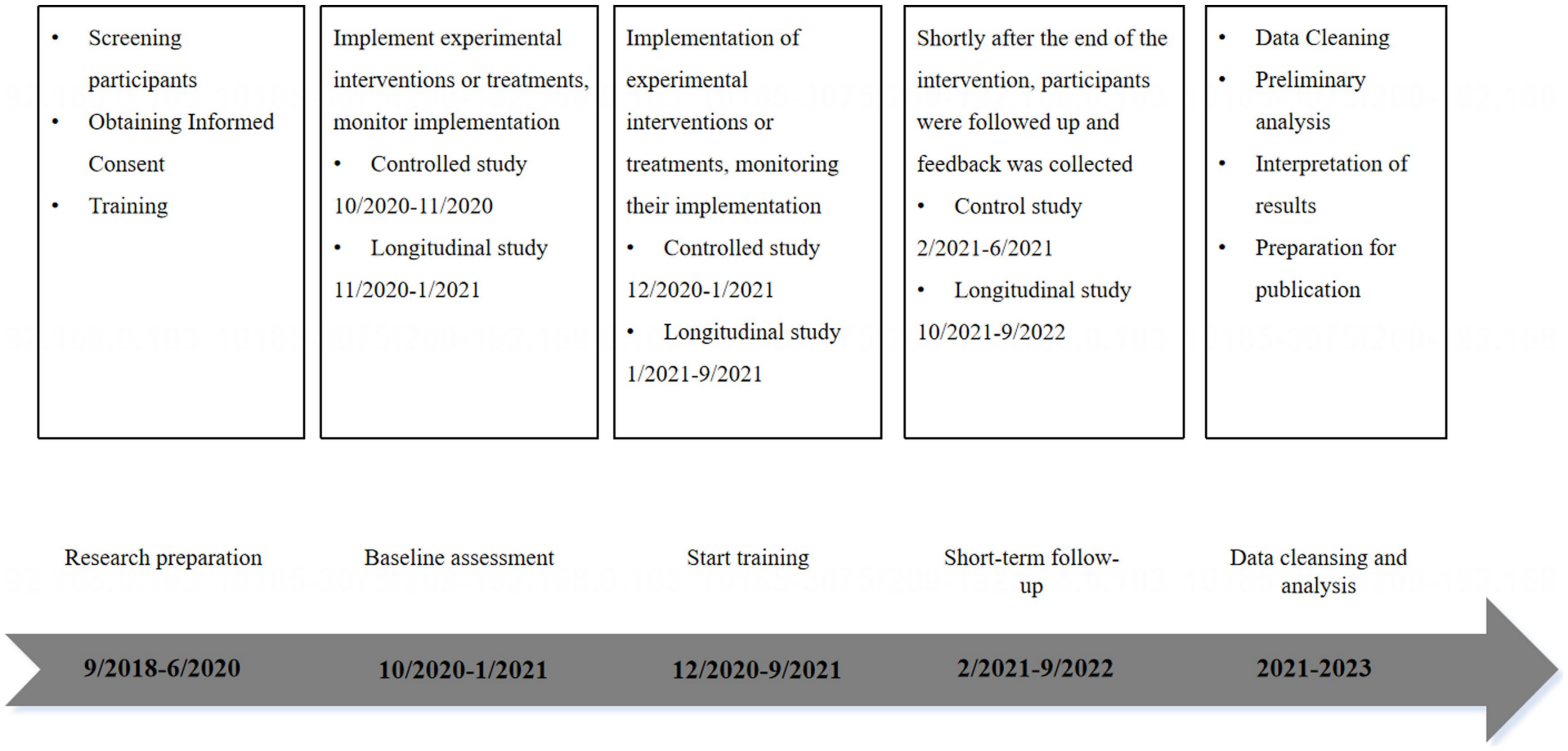
The timeline of cross-sectional and longitudinal study.

This study runs from August 2018 to September 2021 (37 months) with pre-training and mid-intervention completed at the Education and Training Center (Figure 1). Families who came to the center to participate in the intervention experiment applied directly or were referred by a psychiatrist or school teacher. Every participant underwent a series of psychological assessments, such as a hearing examination, the Autism Behavior Scale (ABC) and Children’s Autism Rating Scale (CARS) scales to assess autism, and a clinical interview by the DSM-5 (American Psychiatric Association, DSM-5 Task Force, 2013), followed by diagnosis and supervision by a specialized psychiatrist.

Prior to the start of the experiment, we referred to the effect size of cross-sectional study in Abedi Koupaei et al. (2013) [pre- and post-intervention GARS scores: 55.06 (23.86) vs. 76.47 (15.14), *d* = 1.07], calculated using GPower v3.1 (α = 0.05, Power = 0.8) with a minimum sample size of 30 (15 + 15). Referring to the effect size of Rabeyron et al. (2020) 8-month longitudinal music therapy with 19 children with ASD [pre- and post-intervention CARS scores: 39.3 (7.7) vs. 35.9 (8.2), *d* = 0.97 calculated assuming *r* = 0.5], the minimum sample size calculated using GPower v3.1 (α = 0.05, Power = 0.8) with a minimum sample size of 11.

#### 2.1.1 Inclusion and exclusion criteria

Children with ASD who were included in the study were screened based on the following criteria:

Inclusion criteria: (i) all participants had a diagnosis of ASD according to Diagnostic and Statistical Manual of Mental Disorders [Fifth Edition; DSM-5] from a tertiary care hospital; (ii) the age range was from 3 to 8 years old; (iii) The parents/caregivers agreed to participate in the study and signed an experimental informed consent form; (iv) All included participants did not have specialized musical training or special musical experience; (v) Involvement of a parent or caregiver able to consistently complete assessments throughout the study.

Exclusion criteria: (i) Poor adherence to the study protocol; (ii) Any co-morbid psychiatric or neurodevelopmental disorder or condition that interferes with the subject’s ability to participate in the study or produces an adverse reaction to it, including deafness, or intellectual disabilities according to Diagnostic and Statistical Manual of Mental Disorders [Fifth Edition; DSM-5]; (iii) No response to the TOMATIS test, including no listening curve, no biased lateralization sampling points, and a fit score of 0; (iv) Lifetime history of psychotic disorder; (v) Current or former treatment with cannabinoids.

A total of 90 children, who met the eligibility criteria were initially screened to assign 33 in the cross-sectional study and 57 in the longitudinal study.

In the cross-sectional study, the children were then paired according to their sex, chronological age, and severity of ASD (measured with CARS and ABC). Finally, within each pair, children were randomly assigned to one of two groups by random draw: participants were randomly assigned to the experimental (17) and control (16) groups, who received either 2 sessions of TOMATIS training or intervention training with placebo music over 34 days. The final analyses excluded 2 participants who did not undergo a completed TOMATIS training assessment after random assignment and 1 participant who did not have baseline CARS and ABC scores.

In the longitudinal study, participants received at least 6 phases of intervention training over 7.5 months (90 weeks). Thirty-two participants were excluded because of loss or missing data, and a total of 25 participants ultimately completed the trial. We also recorded the responses and underlying behaviors of the longitudinal study participants when they first took the listening test (see details in Supplementary Table S7).

#### 2.1.2 Baseline evaluations

Of all the participants included in the analysis of intervention effects, the distribution of age groups was as follows: participants ranged in age from 3 to 8 years. The majority of participants (83% in the cross-sectional and 76% in the longitudinal study) were male. Participants in both studies completed all scheduled assessments. Demographic characteristics, baseline scores, symptom severity, and concomitant substance use were similar (Table 1), and none of the subjects had any co-morbid psychiatric or neurodevelopmental disorders.

**Table 1:** Demographic characteristics for the invention and control conditions.

	ASD children in cross-sectional study			ASD Children in the longitudinal study
	Experimental	Control		
	*N* = 15	*N* = 15	p*	*N* = 25
(a) n%				
Primary parent’s education				
High school degree or less	0	0	1	0
3-year junior college	6/15 (40%)	6/15 (40%)	1	2/25 (8%)
4-year college degree or more	9/15 (60%)	9/15 (60%)	1	23/25 (92)
Girls (N)	2/15 (14%)	3/15 (20%)	0.62	6/25 (24%)
Boys (N)	13/15 (86%)	12/15 (80%)		19/25 (76%)
Parents currently married	14/15 (93%)	15/15 (100%)	1	25/25 (100%)
(b) Mean (SD)				
Chronological age (months)	75.6 (32.9)	90.6 (41.8)	0.28	65.6 (25.8)
CARS score	36.5 (4.8)	34.6 (3.2)	0.21	34.9 (3.9)
*N* of Mild ASD (score < 33)	4	6		13
*N* of Moderate ASD (33 < score < 38)	6	7		2
*N* of Severe ASD (Score > 38)	5	2		10
ABC score	105.8 (30.6)	80.4 (16.6)	0.75	——
ER Scores				
Emotion	73.5 (26.2)	71.5 (36.6)	0.99	66.8 (33.5)
Language	101.0 (49.0)	102.0 (55.0)	0.89	108.3 (38.5)
Attention	102.2 (39.1)	100.2 (47.9)	0.74	104.7 (38.6)
Coordination	85.31 (43.0)	75.8 (40.8)	0.5	75.2 (29.7)
SR total scores	52.8 (20.6)	47.4 (23.6)	0.63	59.1 (19.2)

M, mean; SD, standard deviations; N, number of subjects; CARS, Childhood Autism Rating scale; ABC, Autism Behavior Scale. Test statistics and *p-*values reflect group comparisons between Invention and Control conditions. Chi-square tests were not conducted when cell sizes were zero. The sample sizes vary within the group because some demographic data was not provided by some families. * Continuous data was evaluated for *p*-values via the independent samples *t*-test while categorical data was evaluated for intergroup differences via Pearson’s *χ*^2^-test.

In the cross-sectional study, the age of the experimental group was 6.30 ± 2.74 years and the age of the control group was 7.55 ± 3.48 years, there was no significant difference between the two groups, *p* = 0.284; there was no significant difference between the two groups in terms of gender, *p* = 0.624; there was no significant difference between the experimental group and the control group in terms of ABC scores prior to the training, *p* = 0.746, and no significant difference in terms of CARS scores, *p* = 0.211. In the longitudinal experiment, another group of 25 children with ASD (76% male, 24% female) were finally included, with a mean age of 5.47 ± 2.15 years.

### 2.2 TOMATIS trainers and ethical aspects

A total of three researchers and four therapists participated in this study, all four of whom were experienced practitioners specializing in rehabilitation supervision, sensory and special education training, and were accustomed to working with children with ASD. They all regularly communicate verbally, visually/photographically, and gesturally with these stills. In addition, all trainers held qualifications accredited by TOMATIS International development (TDSA Level 4), had completed a specialized TOMATIS Methods training course including theoretical studies and practical exercises, and were required to attend annual advanced training and revalidation to maintain their professional qualifications. After agreeing to participate in the study, they were randomly assigned to either the experimental or control group.

The ethics review committee of Department of Psychology, Renmin University of China reviewed and approved this study. Parents of all children gave written informed consent. The children were recruited through outpatient psychiatry and schools for children with special needs. All were diagnosed by child psychiatrists and for the purpose of this study, their diagnoses were confirmed by child psychiatrists.

### 2.3 Interventions

#### 2.3.1 Training sessions

This study included a cross-sectional experiment as well as a longitudinal experiment. The cross-sectional experiment employed a double-blind randomized controlled study design, ensuring that trainers, parents, and children in both the experimental and control groups remained oblivious to the disparities in intervention methods between the two groups; the intervention was administered by four proficient TOMATIS trainers during each training session.

The cross-sectional study encompassed brief training sessions for children in both groups, spanning 34 days. Prior to the start of the intervention, the researchers held a meeting for the parents of the experimental group, where the treatment process and the purpose of the study were explained to the parents, and the Childhood Autism Rating Scale (CARS) and the Abnormal Behavior Checklist (ABC) were distributed to the children in the experimental group and the control group. The TOMATIS method, as advised by the American Academy of Pediatrics (AAP), involves repeating the 10-day cycle multiple times during the intervention, incorporating 10 days of training in period 1, 7 days of rest, followed by a second period of 10 days of training, and concluding with 7 days of rest after the training, resulting in a total of 2 periods. The researchers created two 10-day training sessions and 7 days of rest for the children in the experimental group. For the duration of the training, music had to be heard 1–2 times a day for 1–2 h, with each session lasting 10 days to guarantee that each child had a minimum of 30 h and a maximum of 40 h of practice. The experimental group was TOMATIS trained, listening to 4 different pieces of music per day. The program of tracks listened to by each participant was personalized according to the participant, with settings for the percentage of sounds of different frequencies, the strength of air conduction, bone conduction, and the processing time delay. The control group listened to the same music as the experimental group but without any processing of parameters such as frequency. Training was not interrupted in both the experimental and control groups.

The control group passively listened to the same repertoire as the experimental group but used a normal Bluetooth headset, which was not affected by the TOMATIS parameter through the Talksup program manager v6.0.2 program, so the trainer did not know whether the parameter being set was for the experimental or control group, and the cell phone could be connected to the TOMATIS programming platform for listening, accompanied by a parent or a trainer to ensure that the music was listened to in its entirety. A parent or trainer will accompany the child during the training to ensure that the child listens to the music in its entirety. The trainer will check the completion of the training and record feedback through online meetings or face-to-face.

The longitudinal study spanned over 7.5 months, with more than 6 sessions, each lasting 10–15 days of training and 2–4 weeks of rest in between. We compared the audiometric core indexes before and after the training and also collected the CARS assessment form at the end of the training. We did multiple regression analyses between the CARS form scores and the audiometric core indexes. During the intervention, parents collaborated to complete the Parent Ten Program Self-Assessment Form and to monitor the initial state of the research, all other training sessions were conducted, resulting in a total of over 6 training sessions being gathered. Parent self-assessment forms were filled out throughout the pre-training initial phase, the intervention phase during training, and the maintenance phase after training.

To ensure consistency and fidelity of the intervention protocol, we trained all trainers in standardized practices prior to the start of the study to ensure consistency in their understanding and implementation of the study protocol. In addition, we implemented regular fidelity checks throughout the intervention, including randomized observation of treatment sessions and assessment of therapist operations to ensure strict adherence to the study protocol.

#### 2.3.2 TOMATIS program

TOMATIS programming follows the seven steps: initial assessment, parent interview, individualized training program design, training, breaks, wrap-up, and feedback, in which the trainer monitors and supports the entire process and the parents are involved in the entire training process. Individualized programs are designed based on each parent interview, hearing test, and training feedback record. Participants were initially given a comprehensive hearing assessment, including measures of sensitivity and responsiveness to different frequencies of sound.

The TOMATIS training program employs gating as a means to regulate the sound frequency entering the middle and inner ear, complemented by lateralized adjustments in the left and right ears, to engage the trainee’s auditory system. The amount of time spent listening is determined by the participant’s habits and adherence. Every session comprised of a preliminary warm-up and a structured training session, known as the listening session (refer to https://advancedbrain.com/ for further information). The goal of this was to give the listener the opportunity to get used to the music before achieving the best level of sensory stimulation. The listening program was composed of three stages: being open to the music (passive acceptance of the music), taking breaks to integrate it, and expressing oneself (listening to the music while practicing vocally). The initial 10-day program primarily comprises Mozart sonatas and other classical compositions, employing music that is softly progressive, typically abundant in harmonics and high frequencies that stimulate the parasympathetic nerves, and can be adjusted to enhance the distinct frequencies of the sounds using specialized TOMATIS headphones; the subsequent 10-day intervention procedure mirrors the initial one, albeit with the distinction that it incorporates 30 min of sound connection, each accompanied by a microphone, to enhance vocal control through self-listening. The study also excluded children who failed to meet the training criteria. Participants were found to have weak areas of auditory frequency based on scores from psychological tests and clinical interviews, and individual music sessions were organized based on the number of musical pieces or chants filtered and the length of the program. The age, diagnosis, duration, and substance of the listening programs received by the retained participants were relatively alike.

The TOMATIS audiometric study process utilized the Madaule and Belfrage (1984) scoring system to assess the eight primary parameters, yielding individual scores spanning from 0 to 10 and composite scores ranging from 0 to 80. Hearing threshold, curve slope, homogeneity, parallelism, balance, spatiality, selectivity, and dominance were among the 8 main parameters that were indexed.

Furthermore, this research modified the assessment criteria. In this study, only the longest time interval data from the initial treatment was utilized when comparing multiple time intervals. Furthermore, excluding the scale data, the scores were modified to account for directionality to align with the notion that “greater scores signify more significant progress,” and the coding of the data was verified by two researchers.

### 2.4 Behavioral assessments

The Childhood Autism Rating Scale (CARS) (Schopler et al., 1988) was designed to assess a child’s communication, activity, intelligence, and overall impression, with a score of 1–4 indicating normal to severely abnormal behavior (Supplementary Table S1). The scale includes verbal, perceptual, and social responses.

The Autism Behavior Scale (ABC) (Krug et al., 1980) comprised 57 items encompassing sensory, interactional, somatic, verbal, and self-care (Supplementary Table S2). The internal reliability, predictive validity, and construct validity of both scales are commendable (Chu et al., 2022).

The Parent Ten-Item Self-Assessment Scale (Supplementary Table S4) was designed to assess the impact of the TE intervention on various aspects, including aspects of motor skills, attention, speech, and social responses. Parents assessed each item on a 10-point scale, with the highest score reflecting the effectiveness of the training.

### 2.5 Assessments of TOMATIS

The TOMATIS training process exclusively utilizes authorized TOMATIS methods and procedures. The TE Listening test device is an AD226 model audiometer manufactured by Danish Hearing International, while the training device is a TOMATIS Development Luxembourg TALKSUP product comprising a mainframe computer and bone conduction headset, along with the program editing software Program Manager LEVEL 4 (Agnew & Associate). The TALKSUP device encompasses all the technological components employed in the TOMATIS approach. The TALKSUP device integrates all the technologies employed in the TOMATIS technique, encompassing the Electronic Ear (EE), comprising an amplifier, a filter, and an electronic gate, primarily designed to “filter the sound” [see details in the flowchart of the EE of Józwiak et al. (2018)]. The Digital Filter System’s Design for Stimulating with Tomatis Method. The process of electronic gating entails modifying the filter type by the level of the signal. The C1 filter is activated when the amplitude level falls below a specific threshold, and it transitions to the C2 filter once it surpasses that threshold. In theory, this abrupt alteration generates a neural stimulus that activates the brain. The C1 filter and C2 filter consist of shelving filters designed for 1 kHz crossband frequencies ranging from −5 to 5 dB, while the electronic gating system in TALKSUP enables real-time processing of music and speech.

TOMATIS consists of both active vocalization and passive listening training, the main difference between the two is that active vocalization training uses the recording material of the child’s voice. Personalized parameter settings include passive listening parameter settings and active listening parameter settings. The passive parameters include the duration, bone conduction delay (D), air conduction delay (P), frequency range, the slope of cut-off frequency, and channels C1 and C2 for sound dynamic filtering for each music track; the active parameters are language-specific frequency parameters (chosen to be set as Chinese frequency-suitable due to the training in a Chinese environment).

### 2.6 Statistical analyses

Repeated measures ANOVA and related samples t-test to test the main effects of group (experimental vs. control) and time (T0, T1) in terms of behavior and audiometric ability. In addition, we did a regression analysis of the core TE listening curve indicators with the scores of the items of the CARS scale to examine the association between the audiometric indicators and the improvement of the external behavior of children with ASD. In this way, we were able to examine the link between the dependent and independent variables.

We also conducted *post-hoc t*-tests to perform comparisons of behavioral and audiometric data over time for each group of children. We also supplemented the results with calculations of Cohen’s *d* for *t*-test effects and $\eta_{p}^{2}$ for *F*-test effects at the current sample size, and simple effects are described with the corresponding estimates and confidence intervals.

## 3 Results

### 3.1 Cross-sectional study

#### 3.1.1 Behavioral improvements

In contrast to the control group, children with ASD showed notable enhancements in auditory status and behavioral symptoms both before and following TOMATIS training (Table 2). Supplementary Table S6 provides comprehensive information on the pre- and post-intervention data for each participant in the experimental group.

**Table 2:** Core indexes of audiometric curves of the experimental and control groups before and after training.

Frequency band	Group	Pre	Post	Percentage improvement	*t*	*p*	Cohen’s *d*
		(M, SD)	(M, SD)				
LF	Experimental	6.00 (1.77)	6.53 (1.36)	14.29%	−2.086	0.056	0.54
MF	Experimental	5.51 (1.54)	7.29 (1.08)	38.15%	−6.403^***^	0.000	1.66
HF	Experimental	4.63 (1.76)	6.11 (1.76)	43.86%	−4.669^***^	0.000	1.21
LHT	Experimental	4.97 (1.55)	6.77 (1.31)	43.23%	−6.158^***^	0.000	1.59
LF	Control	6.40 (1.60)	6.47 (1.19)	3.56%	−0.269	0.792	0.07
MF	Control	6.20 (1.23)	5.99 (1.22)	−3.18%	1.677	0.116	0.43
HF	Control	5.07 (1.47)	5.07 (1.40)	2.24%	−0.041	0.968	0.00
LF	Control	5.68 (1.11)	5.29 (1.06)	−5.91%	1.778	0.097	0.47

M, participant’s average score on the frequency band; SD, standard deviation of participants’ scores in different frequency bands; *p < 0.05, **p < 0.01, ***p < 0.001; LF, low-frequency band; MF, mid-to-high frequency band; HF, high-Frequency band; LHT, lateralization, and hearing thresholds (Same as below). The percentage of improvement in each component was calculated as (post-experiment-pre-experiment)/pre-experiment × 100%.

The 2 (experimental, control) × 2 (pre-training, post-training) repeated measures ANOVA on CARS scores revealed a significant difference between pre- and post-training CARS scores, *F*(1, 28) = 37.95*, p* < 0.001, $\eta_{p}^{2}$ = 0.575. The between-groups differences were not significant, *F*(1, 28) = 0.22, *p* = 0.641. The interaction was significant, *F*(1, 28) = 43.33, *p* < 0.001, $\eta_{p}^{2}$ = 0.607. Simple effects analyses showed that there was no significant difference in CARS scores between the groups before training, *p = 0.*211, and that there was a significant difference in CARS scores between the groups after training, *p* = 0.047, Cohen’s *d* = 0.76, which means that the experimental group’s symptoms of autism were significantly more likely to be improved than the control group after training. Improvement. A significant reduction in CARS scores was observed in the experimental group from pre- to post-training, indicating a substantial effect of the intervention (*p* < 0.001, Cohen’s *d* = 1.42).

The 2 (experimental, control) × 2 (pre-training, post-training) repeated measures ANOVA of ABC scale scores revealed a significant difference in ABC scores between pre- and post-training, *F*(1, 28) = 89.54*, p* < 0.001, $\eta_{p}^{2}$ = 0.762. The between-group effect was not significant, *F*(1, 28) = 2.48, *p* = 0.126. the interaction was significant, *F*(1, 28) = 100.22, *p* < 0.001, $\eta_{p}^{2}$ = 0.782. Simple effects analysis showed that there was no significant difference in ABC scores between the groups before training, *p* = 0.746; the difference in ABC scores between the groups after training was significant, *p* = 0.009, Cohen’s *d* = 0.99, indicating that there was a significant improvement in autism symptoms in the experimental group after training compared to the control group. There was a significant difference in scores before and after training in the experimental group, *p* < 0.001, Cohen’s *d* = 1.69, with a reduction in autism symptoms; there was no significant difference in the control group before and after training, *p* = 0.701 (Table 2).

#### 3.1.2 Improvement of listening capacity

**Figure 2:**
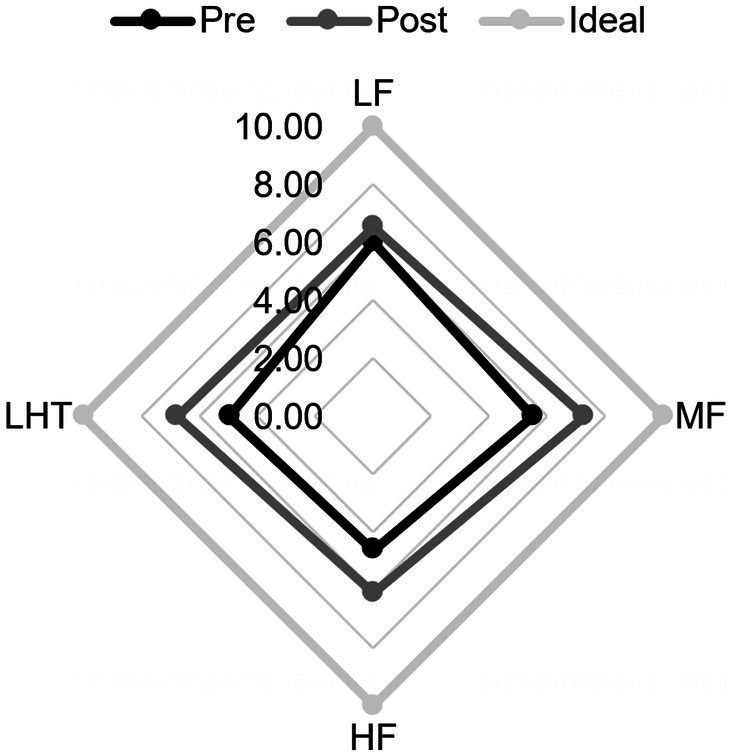
Comparison of audiometric curve indexes before and after training in the experimental group. Pre, pre-training; Post, post-training. LF, low frequency band; MF, mid-to-high frequency band; HF, high Frequency band; LHT, lateralization and hearing thresholds.

**Figure 3:**
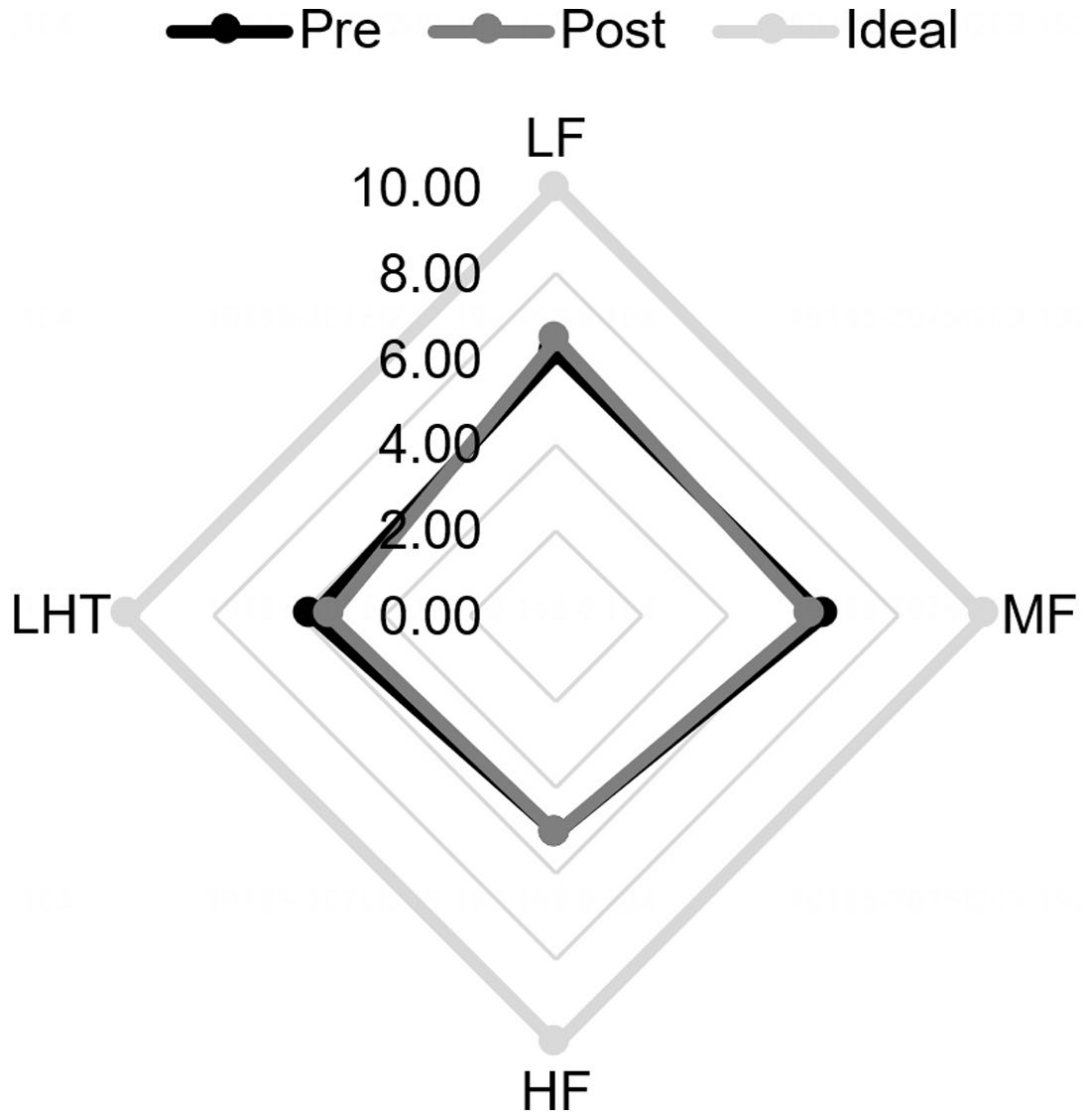
Comparison of audiometric curve indexes before and after training in the controlled group. Pre, pre-training; Post, post-training. LF, low frequency band; MF, mid-to-high frequency band; HF, high Frequency band; LHT, lateralization and hearing thresholds.

Analysis of the TOMATIS listening ability curves revealed that, compared to the control group, participants in the experimental group had better quality of completion at the end than in the first training period after 2 training periods, participants’ cooperation increased, audiometric curve index scores improved, left- and right-ear curve balance improved and became more symmetrical, the slopes of the curves in the frequency bands flattened, spatial localization errors decreased, and left- and right-ear laterality was reduced. The repeated-measures ANOVA revealed significant improvements in scores following mid- and high-frequency training within the experimental group, demonstrating the intervention’s effectiveness, *t* = −6.403, *p* < 0.001, Cohen’s *d* = 1.66; a significant difference in elevation before and after high-frequency training, *t* = −4.669, *p* < 0.001, Cohen’s *d* = 1.21; and a significant difference in elevation before and after mid- and high-frequency training, *t* = −4.669, *p* < 0.001, Cohen’s *d* = 1.21. A significant difference in pre- and post-lift, *t* = −6.158, *p* < 0.001, Cohen’s *d* = 1.59 (Figures 2, 3).

**Figure 4:**
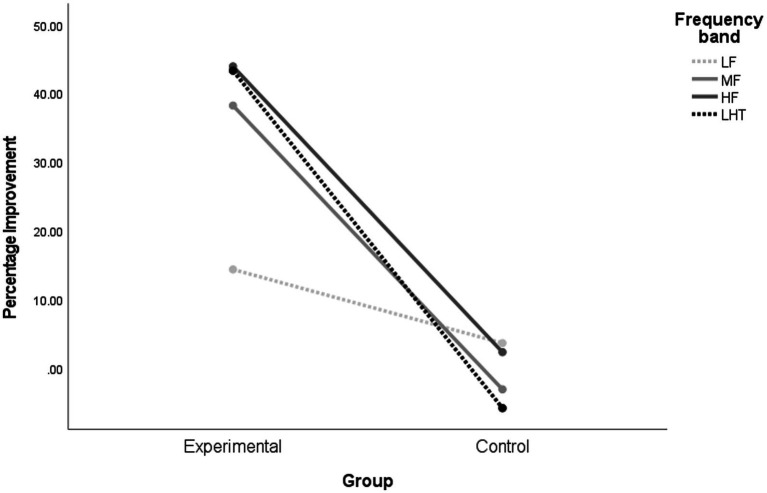
Comparison of the improvement of audiometric indexes in different frequency domains in each group after training. LF, low frequency band; MF, mid-to-high frequency band; HF, high Frequency band; LHT, lateralization and hearing thresholds. Experimental, intervention group; Control, Control Group.

Repeated-measures ANOVA was performed for the percentage improvement of audiometric indices at different frequencies in the experimental group posttest (Figure 4 and Table 2). Pairwise comparisons of different frequencies in the experimental group revealed that improvements in middle and high frequencies, laterality, and hearing thresholds were significantly better than those in low frequencies, *p* < 0.001; there were no significant differences in the percentages of improvement in the other frequency bands.

#### 3.1.3 Correlates of behavioral improvement

There is an inverse relationship between high frequencies and imitation (words and movements), visual response, and somatic usability, indicating enhanced imitation and movement-related behaviors with elevated audiometric indexes, as well as bias versus. There is an inverse relationship between threshold and emotional response, verbal communication, and high activity levels, indicating enhanced emotional and verbal-related behavioral deficits with a greater inclination towards bias. The point at which the audiometric curve reaches its threshold (Table 3).

**Table 3:** Regression analysis of CARS items and audiometric core indicators in the experimental control group.

Items	Frequency band	*B*	SE *B*	β	*t*	*p*	Adjusted *R*^2^	*F*
General impressions	MF	−0.40	0.09	−0.72	−4.55	0.000	0.26	11.15^***^
	HF	0.17	0.08	0.36	2.29	0.026		
Peculiarities of auditory responsiveness	MF	−0.23	0.06	−0.45	−3.82	0.000	0.19	14.55^***^
Imitation	LF	0.13	0.06	0.28	2.17	0.034	0.19	7.75^*^
	HF	−0.20	0.05	−0.49	−3.87	0.000		
Peculiarities of visual responsiveness	HF	−0.15	0.05	−0.39	−3.18	0.002	0.13	10.10^*^
Intellectual functioning	MF	−0.22	0.07	−0.38	−3.14	0.003	0.13	9.86^*^
Inappropriate affect	LF	0.16	0.05	0.41	3.03	0.004	0.13	5.34^*^
	LHT	−0.13	0.06	−0.33	−2.42	0.019		
Anxiety reaction	MF	−0.26	0.08	−0.53	−3.06	0.003	0.12	4.84^*^
	HF	0.13	0.07	0.30	1.77	0.082		
Bizarre use of body movement and persistence of stereotypes	HF	−0.14	0.05	−0.35	−2.84	0.006	0.11	8.05^*^
Resistance to environmental change	LF	0.14	0.05	0.37	2.74	0.008	0.10	4.22^*^
	MF	−0.10	0.06	−0.25	−1.91	0.062		
Verbal communication	LHT	−0.17	0.07	−0.32	−2.54	0.014	0.09	6.47^*^
Activity level	LHT	−0.12	0.06	−0.26	−2.02	0.048	0.05	4.09^*^
Nonverbal communication	MF	−0.13	0.07	−0.24	−1.87	0.066	0.04	3.51
Impairment in human relationships	LHT	−0.09	0.05	−0.22	−1.75	0.086	0.03	3.06
Peculiarities in relating to nonhuman objects	MF	−0.08	0.05	−0.20	−1.59	0.118	0.03	2.52
Total CARS	MF	−1.94	0.33	−0.61	−5.85	0.000	0.36	34.18^***^

*p < 0.05, **p < 0.01, ***p < 0.001; LF, low-frequency band; MF, mid-to-high frequency band; HF, high-Frequency band; LHT, lateralization, and hearing thresholds.

### 3.2 Longitudinal study

#### 3.2.1 Behavioral improvements

**Figure 5:**
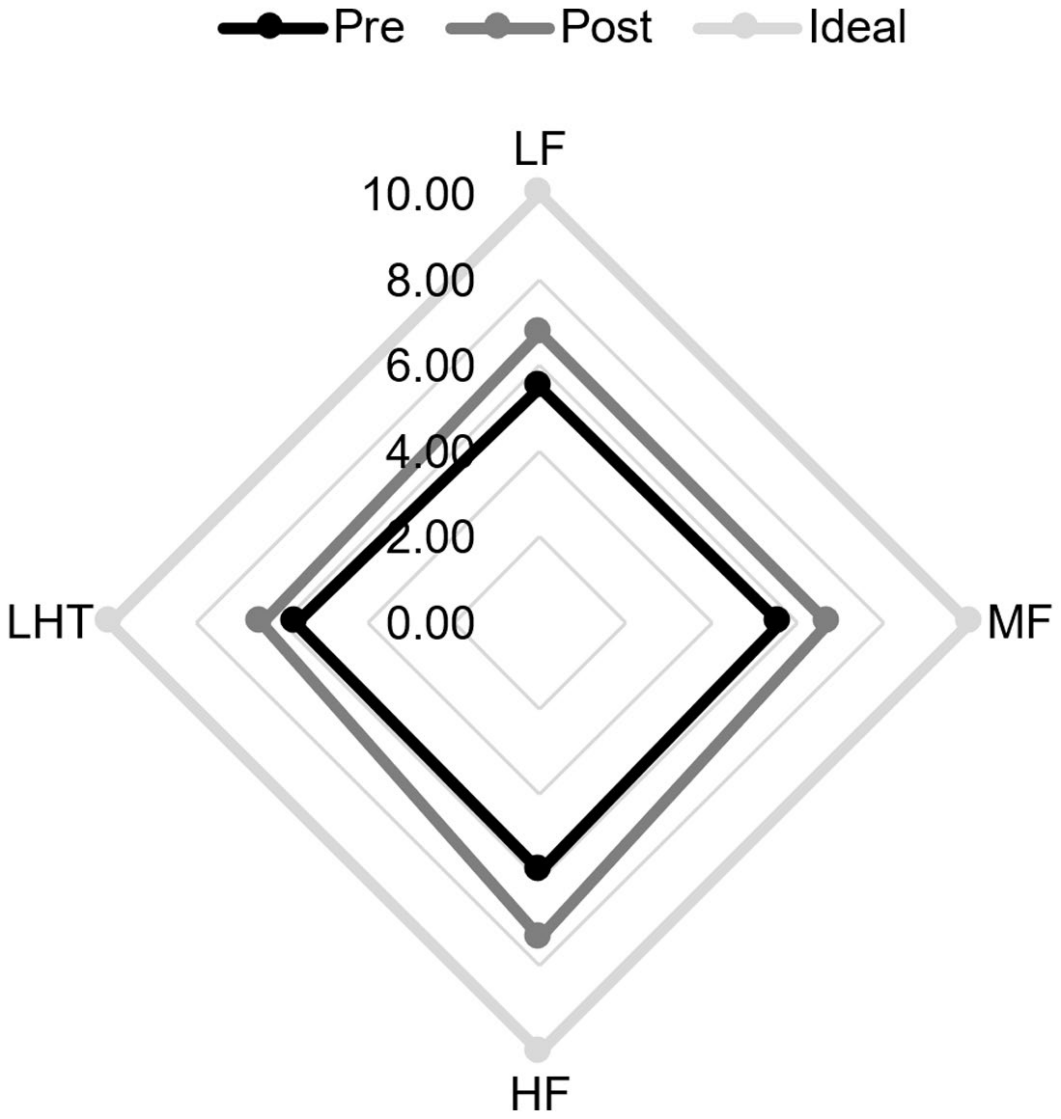
Comparison of audiometric curve indicators before and after longitudinal study training. Pre, pre-training; Post, post-training. LF, low frequency band; MF, mid-to-high frequency band; HF, high Frequency band; LHT, lateralization and hearing thresholds.

Paired-sample *t*-tests showed that comparing the differences in the different frequency domains (Table 3 and Figure 5). There was a significant difference in the pre-and post-training boost in low frequencies, *t* = −2.463, *p* < 0.05, Cohen’s *d* = 0.47; Before and following mid- and high-frequency training, a notable disparity in elevation was observed, with *t* = −2.572, *p* < 0.05, Cohen’s *d* = 0.51; prior to and following high-frequency training, *t* = −3.006, *p* < 0.05, Cohen’s *d* = 0.64; and before and following lateralization and auditory threshold training, *t* = −2.648, *p* < 0.05, Cohen’s *d* = 0.36. The value of Cohen’s d is 0.36, with a *p*-value of less than 0.05 and a value of 2.648. Over the course of the longitudinal period, there was a consistent upward trend in the total scores of all participants, with children diagnosed with autism displaying notable progress in the areas of coordination, language, socialization, and attention. Additionally, the extraction of parent 10-item self-scores from the odd-numbered longitudinal period was conducted for all participants (Supplementary Table S6 and Table 4).

**Table 4:** Core indicators of the audiometric curve of longitudinal participants before and after training.

Frequency band	Group	Pre	Post	Percentage Improvement	t	*p*	Cohen’s *d*
		(M, SD)	(M, SD)				
LF	Experimental	5.49 (2.26)	6.77 (1.35)	114.92%	−2.463^*^	0.039	0.54
MF	Experimental	5.59 (1.87)	6.70 (1.19)	59.77%	−2.572^*^	0.033	1.66
HF	Experimental	5.79 (1.99)	7.34 (0.94)	69.51%	−3.006^*^	0.017	1.21
LHT	Experimental	5.68 (1.66)	6.47 (0.93)	37.96%	−2.648^*^	0.029	1.59

M, participant’s average score; SD, standard deviation of participants’ scores; *p < 0.05; LF, low-frequency band; MF, mid-to-high frequency band; HF, high-Frequency band; LHT, lateralization, and hearing thresholds.

#### 3.2.2 Improvement of listening ability

The negative correlation between HF and interpersonal relationships, total impression, intellectual functioning, verbal communication, imitation (words and actions), adaptation to environmental changes, relationship with inanimate objects, nonverbal communication, visual response, emotional response, and total CARS scores was significant, with improvement in the above behaviors due to elevation of HF metrics; and the negative correlation between LF and proximal sensory response scores was significant, with improvement in proximal sensory response due to elevation of LF metrics (Table 5).

**Table 5:** Regression analysis of each CARS indicator against core listening indicators.

Items	Frequency band	*B*	SE *B*	β	*t*	*p*	Adjusted *R*^2^	*F*
Impairment in human Relationships	HF	−0.63	0.10	−0.82	−6.14	0.000	0.65	37.67^***^
General impressions	HF	−0.55	0.09	−0.81	−6.00	0.000	0.64	35.98^***^
Intellectual functioning	HF	−0.65	0.12	−0.77	−5.23	0.000	0.57	27.36^***^
Verbal communication	HF	−0.42	0.10	−0.70	−4.33	0.000	0.47	18.72^***^
Imitation	HF	−0.40	0.09	−0.70	−4.27	0.000	0.46	18.22^***^
Resistance to environmental change	HF	−0.31	0.08	−0.66	−3.86	0.001	0.41	14.91^*^
Peculiarities in relating to nonhuman objects	HF	−0.29	0.09	−0.58	−3.12	0.006	0.30	9.70^*^
Nonverbal communication	HF	−0.27	0.09	−0.58	−3.07	0.006	0.30	9.41^**^
Peculiarities of visual responsiveness	HF	−0.28	0.11	−0.50	−2.51	0.021	0.21	6.29^*^
Near receptor responsiveness	LF	−0.09	0.04	−0.48	−2.40	0.027	0.19	5.76^*^
Inappropriate affect	HF	−0.26	0.11	−0.47	−2.30	0.033	0.18	5.31^*^
Bizarre use of body movement and persistence of stereotypes	LHT	−0.19	0.10	−0.41	−1.96	0.065	0.12	3.83
Peculiarities of auditory responsiveness	MF	−0.06	0.05	−0.30	−1.37	0.187	0.04	1.88
Activity level	HF	−0.10	0.08	−0.28	−1.26	0.223	0.03	1.58
Anxiety reaction	LHT	−0.04	0.08	−0.12	−0.50	0.621	−0.04	0.25
Total CARS	HF	−4.45	0.71	−0.82	−6.25	0.000	0.66	39.00^***^

*p < 0.05, **p < 0.01, ***p < 0.001; LF, low-frequency band; MF, mid-to-high frequency band; HF, high-Frequency band; LHT, lateralization, and hearing thresholds.

## 4 Discussion

The TOMATIS method uses pure tone detection to determine the frequency profile of the trainee’s auditory counterparts and customizes the program to address the reflected frequency issues. Unlike other sensory integration training, TOMATIS advocates that auditory competence is a process ranging from passive adaptation to active participation, and therefore places greater emphasis on the concept of listening, in which psychomotivation plays a very important role.

The TOMATIS method has not been well researched and applied in children with ASD, and the current discussion on the effectiveness of the method is confusing. Some studies have not found effective results. Corbett et.al (2008) conducted yielded inconclusive findings, potentially attributed to the diverse nature of children with ASD resulting from the limited sample size, as well as the potential utilization of an unsuitable crossover design with enduring advantages in Corbett’s research. The continued observation of statistically significant results in the present study, even after randomization to subgroups, indicates the effectiveness of TOMATIS to some extent. Incorporating TOMATIS into applied interventions could be a viable option. Timely intervention is essential for the formation and operation of the brain. Besides, several previous studies have shown that TOMATIS has a positive effect on attention, sleep, verbal communication, and motor skills (Coppola et al., 2015), and also reduces atypical behaviors (Abedi Koupaei et al., 2013), which is consistent with our findings.

In our cross-sectional study, sensory-motor, attention, social, and emotional processing in children with ASD improved after the TOMATIS intervention. In addition, the experimental group exhibited notable enhancements in the middle and high frequencies, high frequencies, lateralization, and hearing thresholds. Mojs et al. (2011) proposes that the enhancement in these metrics is linked to the auditory hypersensitivity parameters of ASD, hindering the development of children’s social functioning by preventing them from isolating valid social information from auditory disturbances. Conversely, TOMATIS enhances the cochlea’s frequency selectivity through training, enabling the child to identify the “right band” of socially relevant auditory stimuli (Brbić and Tomić, 2020). The study by Kawakami et al. (2020) found that children with ASD have cumulative visual–auditory integration difficulties and attention deficits that may contribute to the core cognitive and social symptoms of ASD.

Through multiple regression analyses, we analyzed in depth the correlations between the frequency bands of hearing indicators and the behavioral indicators of the CARS scale. The low-frequency region primarily pertains to balance, motor skills, and nutritional functions. In physiological structure, the vestibular perceives certain low frequencies that reverberate throughout the body. The TOMATIS Auditory Metrics measure in Pauley’s (2007) research demonstrates attention towards auditory stimuli. Enhanced cognitive processing of the environment and stimulus interactions were linked to the middle and high-frequency bands, while improved imitation and movement were associated with the high frequencies. The cochlea scrutinizes every frequency, with a particular emphasis on the high frequencies (Dallos, 1985; Durrant et al., 2022). Repeated use of high-frequency sounds stimulates the development of middle ear muscles, resulting in better auditory discrimination, clearer attention to stimuli, and better environmental interaction. The cochlear region, which receives high frequencies, is also the first structure to develop in the fetus during pregnancy (Vervoort et al., 2008).

The majority of human speech falls within the frequency range of 500–4,000 Hz, encompassing the mid- and high-frequency bands, with the frequency range of 1,000–3,000 Hz serving as a benchmark for speech and communication. Improvements in language in children with ASD may derive from the fact that acoustic stimuli tend to rely on the auditory system as well as the interconnections between the two hemispheres of the brain to accomplish processing. In general, individuals, because the right ear-laryngeal pathway is shorter than the left ear, speech is delayed by 0.03 s when feedback is dominated by the left ear (Jones et al., 2020; Key and D’Ambrose Slaboch, 2021). Thus, better lateralization and localization mean improved speech.

In addition, emotional responses, language comprehension, and socialization skills are associated with lateralization and auditory thresholds. The frequency range of 2,800 Hz to 3,200 Hz conveys information regarding rhythmic coloration. The melodic, tonal, or rhythmic expression of Mozart’s music may reduce abnormal firing in the brain (Lin et al., 2010). Frequencies exceeding 3,000 Hz are primarily linked to associations and thoughts, and sounds within this frequency spectrum resonate with the head. Besides, The TOMATIS method uses both bone conduction and air conduction for listening. In terms of conduction mechanisms, the voice of the self tends to be transmitted via bone conduction when communicating with the self, while communication with others generally receives sound stimuli via air conduction (Stenfelt and Goode, 2005; Wang et al., 2022). Children with ASD have a high bone conduction predominance and sound localization errors in listening ability assessments; high bone conduction means that participants are primarily listening to their voices and are poor at noticing and communicating with others.

Upon completion of the training, it was evident that the children with ASD exhibited a progressive inclination toward right-ear hearing, and as binaural localization errors diminished, the children with ASD displayed a more substantial enhancement in communication and emotional communication with others. Rauscher et al. (1995) discovered that Mozart’s music stimulated regions of the brain responsible for fine motor coordination, vision, and other advanced cognitive functions. Yuan et al.’s (2018) study conducted in revealed that cortical reward loops were activated by high frequencies.

In general, TOMATIS music training has the potential to create feedback pathways between the auditory organs, the cerebral cortex, and peripheral nerves, affecting the repair and reorganization of the cerebral cortex and muscular pathways through discriminative training in various frequency bands.

Furthermore, due to the delay in auditory integration, the advantages of TOMATIS might be influenced by variations among participants and by “carry-over effects” (Scarpato et al., 2021). The longitudinal study yielded similar findings to the cross-sectional study, with the present study demonstrating behavioral enhancements in the identical group of children with ASD following an extended treatment period. Simultaneously, the current research discovered that low frequency had a beneficial impact on behavior, while low-frequency audiometric measures exhibited an inverse correlation with behavioral enhancement in the cross-sectional study, which might attributed to the existence of a “carry-over effect.”

## 5 Conclusion

Auditory metrics with low frequencies are associated with sensory responses, mid- and high-frequency bands with improved cognitive processing of the environment and stimulus interactions; high frequencies are associated with improvements in imitation and locomotion; and laterality and auditory thresholds are associated with improved functioning in affective responses, language comprehension, and socialization. Behavioral improvements were attributed to the frequency selectivity of TOMATIS training, whereby the auditory organ-cortical feedback loop in children with ASD is exercised and reorganized to filter out ineffective interferences and focus on valid information in the frequency band.

The innovation of the TOMATIS Method contrast to other music therapies is its use of electronic ear technology to modulate sound frequencies, activating the vestibulo-cerebral system through air and bone conduction, with the possible effect of promoting neuroplasticity. It provides an integrated auditory, speech, and cognitive training program.

## 6 Limitations

This study discusses the improvement of TE on the behavior of children with ASD, but future research is necessary to explore the mechanisms involved in TOMATIS in depth using electrophysiological means. In addition to this, there was a reduction in available participant data due to the loss of some subjects during the intervention period. In the future, the sample and intervention period should be increased, and physiological tests such as auditory evoked potentials, electroencephalography, and MRI should be combined to explore the robust effects of TOMATIS. Secondly, TOMATIS training is mainly for children, and it is prudent to generalize the effects of the training to adults with ASD. The rationale behind TOMATIS music training is not yet fully understood, but the results of this study suggest that the brain’s unique patterns in various frequency bands may be associated with improvements in behavioral disorders. The present study’s finding that TOMATIS improves the behavior of children with ASD suggests that TE has the potential for application and theoretical exploration.

## Data availability statement

The original contributions presented in the study are included in the article/Supplementary material, further inquiries can be directed to the corresponding author.

## Ethics statement

The studies involving humans were approved by Ethics Review Committee of Department of Psychology, Renmin University of China. The studies were conducted in accordance with the local legislation and institutional requirements. Written informed consent for participation in this study was provided by the participants’ legal guardians/next of kin.

## Author contributions

YF: Conceptualization, Data curation, Formal analysis, Methodology, Project administration, Software, Supervision, Validation, Visualization, Writing – original draft, Writing – review & editing. MT: Conceptualization, Data curation, Investigation, Methodology, Writing – original draft. JC: Supervision, Visualization, Writing – review & editing. WC: Funding acquisition, Project administration, Resources, Supervision, Validation, Writing – review & editing. HL: Data Curation, Resoures, Writing – review & editing.
