# Supplementary material for: Improvement of symptoms in children with autism by TOMATIS training: a cross-sectional and longitudinal study
Source: Front Behav Neurosci. 2024 Mar 18;18:1357453. doi: 10.3389/fnbeh.2024.1357453 (PMC10982311; doi:10.3389/fnbeh.2024.1357453)
Supplement: Supplementary file 1 [file Data_Sheet_1.docx]

**Supplementary Table 1 Childhood Autism Rating Scale (CARS)**

The Childhood Autism Rating Scale (CARS), abbreviated as CARS Scale, is intended for professional assessment. Each item on the CARS Scale is scored based on the specificity, frequency, and severity of behavior, ranging from 1 with increments of 0.5 to a maximum of 4, using a 7-point scale. A total score greater than 30 suggests autism, 30-36 indicates mild to moderate autism, and a score exceeding 36 with 5 or more items scoring 3 or higher indicates severe autism.

| Name: Age:  **I. Impairment in Human Relationships.** |  |
| --- | --- |
| Age-appropriate; shows age-appropriate shyness, self-defense, and disagreement. | 1 point |
| Mildly abnormal: Lack of some eye contact, unwillingness, avoidance, excessive shyness, mild defects in responding to the examiner. 2 points | 2 points |
| Moderately abnormal: Avoids people, and requires strong disturbance to elicit a response. | 3 points |
| Severely abnormal: Strong avoidance, child rarely responds to the examiner, only responds with strong interference. | 4 points |
| **II. Imitation (Verbal and Motor)** |  |
| Age-appropriate: Imitation consistent with age. | 1 point |
| Mildly abnormal: Mostly imitates, sometimes excited, sometimes delayed. | 2 points |
| Moderately abnormal: Sometimes imitates under great demands from the examiner. | 3 points |
| Severely abnormal: Rarely imitates language or motor actions of others. | 4 points |
| **III. Inappropriate Affect.** |  |
| Age-appropriate: Emotionally responsive to age and context—happy, unhappy, interested—expressed through facial expressions and gestures. | 1 point |
| Mildly abnormal: Some lack appropriate responses to different emotional stimuli; emotions may be limited or excessive. | 2 points |
| Moderately abnormal: Inappropriate expression of emotions, limited or excessive reactions, or often unrelated to stimuli. | 3 points |
| Severely abnormal: Extremely stereotyped emotional responses; rarely produces appropriate responses in situations where the examiner insists on changes. | 4 points |
| **IV. Bizarre Use of Body Movement and Persistence of Stereotypes.** |  |
| Age-appropriate: Appropriate use and awareness of the body. | 1 point |
| Mildly abnormal: Some peculiarities in body use—stereotyped movements, clumsiness, lack of coordination. | 2 points |
| Moderately abnormal: Signs of moderate special dysfunction in finger or body posture functions, such as rocking, rotating, finger flicking, and tiptoe walking. | 3 points |
| Severely abnormal: Occurs severely and widely as described above. | 4 points |
| **V. Peculiarities in Relating to Nonhuman Objects (like toys and other**  **materials.)** |  |
| Age-appropriate: Appropriate interest, use, and exploration of objects. | 1 point |
| Mildly abnormal: Mild lack or inappropriate use of objects, biting objects like a baby, hitting objects vigorously, being obsessed with the squeaking sound of objects, or constantly turning lights on and off. | 2 points |
| Moderately abnormal: Lack of interest in most objects or somewhat peculiar behaviors, such as repeatedly rotating an object, picking up things with fingertips, rotating wheels, or being fascinated with a particular part. | 3 points |
| Severely abnormal: Severe inappropriate interest, use, and exploration of objects, as described above, frequently occurring, making it difficult to distract the child. | 4 points |
| **VI. Resistance to Environmental Change.** |  |
| Age-appropriate: Adaptive responses to changes consistent with age. | 1 point |
| Mildly abnormal: Some reactions to environmental changes, tending to maintain certain object activities or insist on the same response form. | 2 points |
| Moderately abnormal: Signs of irritability and depression in response to environmental changes, difficult to attract attention when disturbed. | 3 points |
| Severely abnormal: Severe reactions to changes; if environmental changes are imposed persistently, the child may escape. | 4 points |
| **VII. Peculiarities of Visual Responsiveness.** |  |
| Age-appropriate: Age-appropriate visual responses, integrated with other sensory systems. | 1 point |
| Mildly abnormal: Sometimes need to remind the child to pay attention to objects; sometimes completely absorbed in "mirror images"; some avoid eye contact; some stare into space; some are fascinated by lights. | 2 points |
| Moderately abnormal: Often need to remind them of what they are doing; like to watch bright objects; even if forced, there is little eye contact; staring at people or staring into space. | 3 points |
| Severely abnormal: Extensive and severe visual avoidance of objects and people; fascinated by using "peripheral vision." | 4 points |
| **VIII.** **Peculiarities of Auditory Responsiveness.** |  |
| Age-appropriate: Age-appropriate auditory responses. | 1 point |
| Mildly abnormal: Some lack of response to auditory stimuli or certain special sounds; delayed reactions; sometimes sound stimuli need to be repeated; sometimes sensitive to loud sounds or distracted by them. | 2 points |
| Moderately abnormal: No response to auditory stimuli or must be repeated several times for a response; sensitive to certain sounds (easily startled, covering ears, etc.). | 3 points |
| Severely abnormal: Complete avoidance of sounds, no attention to types of sounds, or extreme sensitivity. | 4 points |
| **IX. Near Receptor Responsiveness.** |  |
| Age-appropriate: Appropriate reactions to pain, normal touch, and smell. | 1 point |
| Mildly abnormal: Some lack appropriate response to pain or light touch, smells, tastes, etc.; sometimes show signs of sucking on objects. | 2 points |
| Moderately abnormal: Lack of response to pain or accidental injury, more focused on touch, smell, and taste. | 3 points |
| Severely abnormal: Overly focused on exploring the sense of touch rather than functional use (sucking, licking, or rubbing), completely ignores pain, or reacts excessively. | 4 points |
| **X. Anxiety Reaction** |  |
| Age-appropriate: Responds to situations with age-appropriate reactions, and the reaction does not persist. | 1 point |
| Mildly abnormal: Mild anxiety response. | 2 points |
| Moderately abnormal: Moderate anxiety response. | 3 points |
| Severely abnormal: Severe anxiety response; the child may not sit for some time during a meeting, may be afraid, withdraw, etc. | 4 points |
| **XI. Verbal Communication.** |  |
| Age-appropriate: Age-appropriate language. | 1 point |
| Mildly abnormal: Language is slow, most language is meaningful, but there is some imitation of language. | 2 points |
| Moderately abnormal: Lack of language or meaningful language confused with inappropriate language (imitating speech or speaking inexplicably). | 3 points |
| Severely abnormal: Severely abnormal speech, essentially lacks understandable language or uses special and bizarre language. | 4 points |
| **XII. Non-verbal Communication** |  |
| Age-appropriate: Non-verbal communication consistent with age. | 1 point |
| Mildly abnormal: Non-verbal communication is slow; interactions are simple or vague, such as pointing or going to get what they want. | 2 points |
| Moderately abnormal: Lack of non-verbal communication; the child does not use or respond to non-verbal communication. | 3 points |
| Severely abnormal: Especially strange and incomprehensible non-verbal communication. | 4 points |
| **XIII. Activity Level.** |  |
| Age-appropriate: Normal activity level—neither too active nor too inactive. | 1 point |
| Mildly abnormal: Mildly restless or slightly slow in activity, but generally controllable. | 2 points |
| Moderately abnormal: Activity is quite high, and controlling the level of activity is difficult; or quite inactive or slow in movement, the examiner frequently needs to control or make a great effort to elicit a response. | 3 points |
| Severely abnormal: Extremely abnormal activity level, either non-stop or indifferent; it is difficult to get the child to react to any event, almost constantly requiring adult control. | 4 points |
| **XIV.Intellectual Functioning** |  |
| Age-appropriate: Normal intellectual function—no evidence of dullness. | 1 point |
| Mildly abnormal: Mild intellectual impairment—skills are low in all areas. | 2 points |
| Moderately abnormal: Moderate intellectual impairment—some skills are significantly delayed, while others are close to age level. | 3 points |
| Severely abnormal: Severe intellectual impairment—some skills are significantly delayed, while others are above age level or unusual. | 4 points |
| **XV. General Impressions.** |  |
| Age-appropriate: Not autistic. | 1 point |
| Mildly abnormal: Slight or mild autism. | 2 points |
| Moderately abnormal: Moderate signs of autism. | 3 points |
| Severely abnormal: Very many signs of autism. | 4 points |

**Supplementary Table 2 Autism Behavior Checklist—ABC Scale**

The Autism Behavior Checklist (ABC Scale) is designed to assess various behaviors associated with autism. The scale consists of five subscales, each addressing different aspects of behavior. Professionals use the ABC Scale to gain insights into the presence and severity of behaviors commonly observed in individuals with autism spectrum disorder (ASD). The scores are indicative of the degree of impairment in each domain.

Child's Name: __________________ Date of Birth: __________________

Relationship between the Examiner and the Child: __________________ Date of Assessment: __________________

Scoring:

| Item | Behavior | Answer |
| --- | --- | --- |
| C4 | 1. Enjoys prolonged self-rotation | Yes / No |
| E2 | 2. Learns to do a simple task but quickly forgets | Yes / No |
| A4 | 3. Often avoids contact with the environment or social interaction | Yes / No |
| D1 | 4. Often cannot follow simple commands (e.g., sit down, come here) | Yes / No |
| C2 | 5. Does not play with toys (e.g., endlessly spinning, throwing, kneading) | Yes / No |
| A2 | 6. Poor visual discrimination skills (e.g., difficulty distinguishing features, size, color, position of an object) | Yes / No |
| B2 | 7. Lacks social smiles (i.e., does not nod, greet, smile) | Yes / No |
| D3 | 8. Reverses or confuses pronoun use (e.g., can't distinguish between "you" and "me") | Yes / No |
| C3 | 9. Holds onto something for a long time | Yes / No |
| A3 | 10. Appears not to listen to people speaking, leading some to suspect hearing issues | Yes / No |
| D4 | 11. Speech lacks intonation and rhythm | Yes / No |
| C4 | 12. Swings body for extended periods | Yes / No |
| B3 | 13. Attempts to reach for objects in places the body cannot reach (i.e., poor estimation of distance) | Yes / No |
| E3 | 14. Strong reactions to changes in the environment and daily routines | Yes / No |
| D2 | 15. No response when called by name in the presence of others | Yes / No |
| C4 | 16. Often makes forward rushes, spins, walks on tiptoes, finger flicks, etc. | Yes / No |
| B3 | 17. No response to facial expressions of others | Yes / No |
| D2 | 18. Rarely uses words like "yes" or "me" | Yes / No |
| E4 | 19. Displays a special ability that seems incongruent with low intelligence | Yes / No |
| D1 | 20. Cannot execute simple prepositional instructions (e.g., put the ball on the box or in the box) | Yes / No |
| A3 | 21. Sometimes shows no reaction to loud sounds (may lead to suspicions of deafness) | Yes / No |
| C4 | 22. Often claps hands | Yes / No |
| E3 | 23. Tantrums or frequently displays anger | Yes / No |
| B4 | 24. Actively avoids eye contact with others | Yes / No |
| B4 | 25. Refuses contact or hugs from others | Yes / No |
| A3 | 26. Sometimes shows no reaction to painful stimuli such as falls, cuts, or injections | Yes / No |
| B3 | 27. The body appears very stiff and difficult to hold | Yes / No |
| B2 | 28. Feels relaxed when held, even if not tightly held | Yes / No |
| D2 | 29. Expresses desires through posture and gestures rather than language | Yes / No |
| C2 | 30. Often walks on tiptoes | Yes / No |
| E2 | 31. Engages in biting, hitting, kicking, etc., to harm others | Yes / No |
| D3 | 32. Repeats short sentences over and over | Yes / No |
| B3 | 33. Does not imitate other children during play | Yes / No |
| A1 | 34. Often does not blink when bright light shines directly into the eyes | Yes / No |
| C2 | 35. Engages in self-harming behaviors such as head-banging or hand-biting | Yes / No |
| E2 | 36. Cannot wait for something (wants it immediately) | Yes / No |
| D1 | 37. Cannot name more than 5 objects | Yes / No |
| B4 | 38. Does not develop any friendships (does not interact with other children) | Yes / No |
| A4 | 39. Covers ears when there are many loud noises | Yes / No |
| C4 | 40. Often rotates and collides with objects | Yes / No |
| E1 | 41. Difficulty in toilet training (cannot control bowel movements) | Yes / No |
| D2 | 42. Can only make requests for 5 or fewer things in a day | Yes / No |
| B3 | 43. Often startled or very anxious | Yes / No |
| A3 | 44. Squints, closes eyes, frowns in normal light | Yes / No |
| E1 | 45. Cannot dress independently without help | Yes / No |
| D3 | 46. Repeatedly repeats sounds or words | Yes / No |
| B4 | 47. Stares at people as if trying to see through them | Yes / No |
| D4 | 48. Repeats questions asked by others or repeats answers | Yes / No |
| E2 | 49. Often unaware of the environment and may not pay attention to dangerous situations | Yes / No |

**Supplementary Table 2 TOMATIS Training Sensory SR Adjustment Scale**

The TE (TOMATIS Equipment) comes with specialized assessment scales specifically designed for TOMATIS training. These assessments are suitable for children and adolescents aged 1-18 or individuals with functional abilities within this range. The scales include the Sensory Regulation Assessment (SR) (see Appendix 3) and the Executive Regulation Assessment (ER) (see Appendix 4).

The SR assessment includes nine dimensions: "Self-Regulation," "Attention," "Sleep," "Eating and Oral Movements," "Tactile System," "Vestibular," "Auditory," "Visual," and "Attachment and Emotion." It comprises a total of 143 items, with each item scored on a 10-point scale.

The ER assessment consists of four dimensions: "Emotion," "Language," "Attention," and "Coordination." It includes a total of 80 items, and each item is rated on a 10-point scale. Higher scores indicate more severe issues.

The SR and ER assessments serve as a basis for developing TOMATIS programs and also function as tools to assess the effectiveness of the training.

| **Self-Regulation Aspect** | |
| --- | --- |
| **For each statement, please rate your child on a scale from 0 to 10, where 0 indicates "Not at all" and 10 indicates "Very much."** | **Score** |
| 1. Is your child often irritable? |  |
| 2. Does your child escalate quickly from a good mood to intense anger, even with minimal external stimuli? |  |
| 3. Is it difficult for your child to calm down during relaxation activities, and even when attempting to self-soothe, does your child still feel anxious and restless? |  |
| 4. Can your child not wait calmly for things to happen, displaying impatience? |  |
| 5. Does your child dislike transitioning from one activity to another and prefer to continue doing the same thing once started? |  |
| 6. Does your child require planning before introducing new activities and dislike unpredictable plan changes? |  |
| 7. Does your child rely on others to maintain a state of doing things and find it challenging to work independently? |  |
| 8. Does your child frequently have severe temper tantrums, explosive outbursts, or anger issues, finding it hard to control themselves? |  |
| 9. If your child doesn't have time for calming activities each day, does he or she become very irritable and anxious? |  |
| 10. If your child does many things on the same day, does he or she feel the pace is too fast, causing stress and nervousness? |  |
| 11. Is your child prone to outbursts, yelling, or throwing things? |  |
| 12. Does your child's temper lead to trouble at home or school, affecting relationships? |  |
| 13. Does your child get upset by trivial matters? |  |
| 14. Is your child frequently angry and resentful? |  |
| 15. Does your child often cry easily? |  |
| **Attention Aspect** | |
| **For each statement, please rate your child on a scale from 0 to 10, where 0 indicates "Not at all" and 10 indicates "Very much."** | **Score** |
| 1. Is your child easily distracted, with a short duration of attention? |  |
| 2. Does your child daydream and find it difficult to focus on activities he/she wants to concentrate on? |  |
| 3. Is it challenging for your child to easily shift attention from one activity to another? |  |
| 4. Does your child have difficulty sticking to one idea or goal, often getting off track? |  |
| 5. Is it hard for your child to focus on one thing at a time? |  |
| 6. Does your child feel restless, unable to sit still for an extended period, and very agitated? |  |
| 7. Does your child make careless mistakes or have difficulty paying attention to details? |  |
| 8. Does your child frequently lose important items or place things in the wrong location? |  |
| 9. Does your child have difficulty organizing projects or activities? |  |
| 10. Is it challenging for your child to complete tasks? |  |
| 11. Does your child forget important tasks or often do things late? |  |
| 12. Is your child always busy and occupied? |  |
| 13. Does your child become chaotic during tasks? |  |
| 14. Is it difficult for your child to wait in line or wait for things to happen? |  |
| 15. Does your child act impulsively, taking action before thinking? |  |
| 16. Does your child have messy handwriting? |  |
| 17. Is it hard for your child to wait for instructions before starting a task or difficult to read instructions? |  |
| 18. Does your child have difficulty completing tasks on time, requiring extra time? |  |
| **Sleep Aspect** | |
| **For each statement, please rate your child on a scale from 0 to 10, where 0 indicates "Not at all" and 10 indicates "Very much."** | **Score** |
| 1. Does your child wake up three times or more at night and then have difficulty falling back asleep? |  |
| 2. Does your child have significant trouble falling asleep, taking more than 30 minutes to gradually fall asleep? |  |
| 3. Does your child have nightmares or night terrors? |  |
| 4. Does your child wake up in the morning feeling unrested? |  |
| 5. Does your child always feel tired? |  |
| 6. Does your child find it difficult to fall asleep without white noise (such as an oscillating fan or air conditioning)? |  |
| 7. Does your child have an irregular sleep schedule, going to bed at different times each night? |  |
| **Eating/Oral Movement Aspect** | |
| **For each statement, please rate your child on a scale from 0 to 10, where 0 indicates "Not at all" and 10 indicates "Very much."** | **Score** |
| 1. Does your child prefer foods with a uniform texture and dislike foods with lumps or uneven textures? |  |
| 2. Does your child crave certain foods or drinks? |  |
| 3. Does your child prefer overly spicy, sweet, sour, or salty foods? |  |
| 4. Does your child eat excessively, even when already full? |  |
| 5. Does your child have a big appetite? |  |
| 6. Is your child picky, avoiding trying new foods and only eating specific items? |  |
| 7. Does your child go for long periods without eating but doesn't feel hungry? |  |
| 8. Is your child too restless to sit down and eat? |  |
| 9. Does your child eat when feeling anxious or upset? |  |
| 10. Does your child eat blindly? |  |
| 11. Does your child lose appetite when feeling anxious or upset? |  |
| 12. Does your child still excessively like to have things in their mouth beyond an appropriate age? |  |
| 13. Does your child have difficulty sucking, chewing, and swallowing? |  |
| 14. Is your child easily choked or obstructed by food or liquid? |  |
| 15. Does your child lick, taste, or chew on non-edible objects like pens, straws, shirt sleeves, and others? |  |
| 16. Does your child grind teeth throughout the day? |  |
| 17. Does your child excessively drool during the teething phase? |  |
| **Touch Aspect** | |
| **For each statement, please rate your child on a scale from 0 to 10, where 0 indicates "Not at all" and 10 indicates "Very much."** | **Score** |
| 1. Does your child like certain clothes while finding others uncomfortable, feeling too tight, or itchy? |  |
| 2. Does your child cut labels off clothes? |  |
| 3. Does your child enjoy wearing multiple layers of clothing or very tight-fitting clothes? |  |
| 4. Does your child have difficulty enjoying baths or showers, especially dislikes washing hair and face? |  |
| 5. Does your child dislike haircuts or nail trimming, avoiding hair cleaning or brushing? |  |
| 6. Does your child dislike hugs and physical contact from others? |  |
| 7. Is your child eager to touch everything, constantly bumping into people or objects? |  |
| 8. Is your child unaware of pain, not noticing cuts, or bruises, and feeling nothing with medical procedures or injections? |  |
| 9. Does your child avoid touching certain textures of clothes and materials, and avoid getting hands dirty? |  |
| 10. Does your child seek surfaces or objects with strong tactile feedback? |  |
| 11. Can't determine which part of their body is being touched if not looking? |  |
| 12. Does your child become fearful, anxious, or aggressive with slight or unexpected touches? |  |
| 13. Is your child unaware of touches or collisions unless they are strong and intense? |  |
| **Vestibular Aspect** | |
| **For each statement, please rate your child on a scale from 0 to 10, where 0 indicates "Not at all" and 10 indicates "Very much."** | **Score** |
| 1. Does your child frequently move, being unable to sit still during activities? |  |
| 2. Is your child afraid of swinging or similar movements, scared of amusement park rides, escalators, etc.? |  |
| 3. Does your child dislike heights? |  |
| 4. Does your child crave intense physical activities like swinging, running, and dancing? |  |
| 5. Is your child having difficulty learning new motor skills? |  |
| 6. Does your child enjoy being thrown into the air for a long time or swinging as high as possible, liking "rough" games like wrestling? |  |
| 7. Does your child hit, bump, or push other children? |  |
| 8. Does your child crave vibrations or intense sensory input? |  |
| 9. Does your child have difficulty regulating pressure when writing, walking, or grabbing objects? |  |
| 10. Does your child sway their body, shake their head, or kick their legs when sitting at a desk? |  |
|  |  |
| 11. Does your child slump and appear weak or lazy when working at a desk? |  |
| 12. Does your child sit on the floor in a "W" shape to stabilize their body? |  |
| 13. Does your child tire easily and prefer tasks that can be done sitting? |  |
| 14. Does your child have poor body awareness, often bumping into things, knocking things over, and appearing clumsy? |  |
| 15. Does your child have poor gross motor skills, such as jumping, catching, skipping, climbing ladders, etc.? |  |
| 16. Does your child have difficulty with fine motor skills required for using tools, like scissors, holding a pencil, using utensils, or dressing (buttoning, zipping)? |  |
| 17. Does your child avoid/dislike movement activities like playground equipment (swings, ladders, slides, or merry-go-round), and elevators/escalators? |  |
| 18. Is your child afraid of going up, down, or walking on uneven surfaces like curbs or steps? |  |
| 19. Is your child afraid of being upside down, sideways, or backward, e.g., leaning back while washing hair? |  |
| 20. Is your child afraid of their feet leaving the ground? |  |
| 21. Does your child easily lose balance, appearing clumsy and afraid of activities requiring good balance, like riding a bike, jumping, or a one-legged balance? |  |
| **Auditory Aspects** | |
| **Please rate your child on a scale of 0-10, with decimals allowed, based on the following:** | **Score** |
| 1. Does your child get startled or easily distracted by loud noises such as a vacuum cleaner, sirens, or doorbells? |  |
| 2. Is your child easily distracted by sounds that others might not notice? |  |
| 3. Does your child have difficulty hearing conversations even though their hearing is fine? |  |
| 4. Is your child bothered or distracted by background noises? |  |
| 5. Does your child interrupt others before they finish speaking? |  |
| 6. Is your child more inclined to stay silent rather than talk? |  |
| 7. Does your child talk excessively? |  |
| 8. Does your child seem not to notice when verbally addressed or named? |  |
| 9. Does your child prefer very loud music or television? |  |
| 10. Is your child confused about the source and direction of sounds? |  |
| 11. Does your child mutter or speak loudly while completing tasks? |  |
| 12. Did your child make few or no vocalizations as a baby, such as babbling? |  |
| 13. Does your child need instructions to be repeated many times or frequently ask, "What did you say?" |  |
| **Visual Aspects** | |
| **Please rate your child on a scale of 0-10, with decimals allowed, based on the following:** | **Score** |
| 1. Is your child sensitive to bright lights, squinting, or needing sunglasses? |  |
| 2. Does your child enjoy being in crowded, bustling environments like supermarkets or malls? |  |
| 3. Does your child feel overwhelmed in open spaces? |  |
| 4. Does your child dislike disorder? |  |
| 5. Is your child easily distracted by visual stimuli in a room, such as moving people or objects, decorations, toys, windows, or doorways? |  |
| 6. Does your child struggle to see the whole "big picture," focusing too much on details or patterns in pictures? |  |
| 7. Does your child have difficulty finding things in other items (e.g., papers on a table, clothes in a drawer, items on a grocery shelf, or toys in a box)? |  |
| 8. Does your child have difficulty controlling eye movements to track and follow moving objects? |  |
| 9. Does your child struggle to differentiate between different colors, shapes, and sizes? |  |
| **Attachment/Emotional Functioning** | |
| **Please rate your child on a scale of 0-10, with decimals allowed, based on the following:** | **Score** |
| 1. Does your child avoid eye contact and prefer not to look at others' faces during conversations? |  |
| 2. Does your child feel depressed and rarely experience joy or happiness? |  |
| 3. Is your child not proactive in interacting with others and prefers solitude? |  |
| 4. Does your child find it challenging to fully engage in conversations? |  |
| 5. Does your child lack imagination or have minimal imaginative abilities? |  |
| 6. If upset or angry, does your child engage in destructive behavior, such as breaking things? |  |
| 7. Is it difficult for your child to be separated from family members and be alone? |  |
| 8. Does your child have almost no close or intimate relationships with friends or family? |  |
| 9. Does your child get angry or aggressive towards others, easily having outbursts? |  |
| 10. Is it challenging for your child to set self-limits to prevent doing inappropriate things? |  |
| 11. Does your child struggle with self-discipline when doing things they should do? |  |
| 12. Does your child feel anxious or fearful about new people and situations? |  |
| 13. Is your child heavily burdened by fear/traumatic events? |  |
| 14. Does your child engage in self-harm or self-destructive behaviors? |  |
| 15. Does your child need complete control over the environment, disliking sharing control with others? |  |
| 16. Does your child engage in ritualistic behaviors repeatedly? |  |
| 17. Is your child a high perfectionist? |  |
| 18. Does your child find it difficult to understand others' emotions? |  |
| 19. Is your child rarely invited to social activities such as parties or dates? |  |

**Supplementary Table 3 TOMATIS Training Executive Function ER Adjustment Scale**

| Emotions |  |
| --- | --- |
| **Please rate your child on a scale of 0-10, with decimals allowed, based on the following:** | Score |
| 1. Does your child behave more childishly than their actual age? |  |
| 2. Does your child often argue and get involved in many fights? |  |
| 3. Does your child frequently boast and show off? |  |
| 4. Is it challenging to divert your child's thoughts from certain ideas? |  |
| 5. Is your child cruel or nasty to others or animals, bullying them? |  |
| 6. Does your child have difficulty getting along with other children (getting teased)? |  |
| 7. Does your child have guilt disorders, not feeling guilty after doing something wrong, or always feeling guilty? |  |
| 8. Does your child feel they must be perfect? |  |
| 9. Does your child feel worthless or inferior? |  |
| 10. Is your child in a tense, worried, fearful, anxious, highly sensitive, or tense state? |  |
| 11. Does your child compulsively repeat certain behaviors? |  |
| 12. Is your child timid, shy, embarrassed, or easily awkward? |  |
| 13. Is your child stubborn, gloomy, or irritable? |  |
| 14. Does your child experience sudden changes in mood or feelings during the day? |  |
| 15. Is your child unhappy, sad, or depressed, often or easily crying? |  |
| 16. Does your child withdraw or not participate in activities with others? |  |
| 17. Does your child almost not accept changes in routine? |  |
| 18. Is your child easily frustrated or irritable? |  |
| 19. Does your child perform best in group work or individual work, preferring to observe? |  |
| 20. Is it difficult to calm or self-soothe your child with a pacifier, comfort object, or caregiver? |  |
| **Language** |  |
| **Please rate your child on a scale of 0-10, with decimals allowed, based on the following:** | Score |
| 1. Does your child often speak recklessly or "off-topic"? |  |
| 2. Is it difficult for your child to express, articulate, or pronounce words incorrectly (e.g., similar-sounding words)? |  |
| 3. Is it difficult for your child to perceive and understand what is said in a noisy environment? |  |
| 4. Is it difficult for your child to perceive and understand what is said in a quiet environment? |  |
| 5. Is it harder for your child to understand the meaning when speaking in sentences rather than single words? |  |
| 6. Does your child speak very fast? |  |
| 7. Is it difficult for your child to say lengthy and complex sentences (e.g., "I saw my teacher on the street on my way to school")? |  |
| 8. Is there a speech disconnect or incoherence, making it hard to understand what your child is saying? |  |
| 9. Is it challenging for your child to turn their thoughts into words, tell stories, or describe situations? |  |
| 10. Does your child intermittently interrupt their speech with phrases (e.g., yes, sure, like, okay)? |  |
| 11. Does your child bring the conversation back to their favorite topic without listening to what others want to say? |  |
| 12. Is your child's thinking very concrete, understanding things literally, or finding it hard to grasp humor? |  |
| 13. Is it difficult for your child to understand social rules (e.g., politeness), leading to them getting into trouble, appearing disrespectful, or rude? |  |
| 14. Do social difficulties arise for your child due to a lack of wit in communication (e.g., saying to an elderly person, "Oh, you're old")? |  |
| 15. Is your child unaware that someone is trying to communicate with them, continuing to indulge in their activities? |  |
| 16. Does your child not communicate spontaneously? |  |
| 17. Is it challenging for your child to use body language to express emotions or express emotions inappropriately (e.g., showing a happy expression upon hearing sad news)? |  |
| 18. Is it difficult for your child to understand the meaning of body language and facial expressions in social situations? |  |
| 19. Does your child look at others but not truly see them, seeming distracted during communication? |  |
| 20. Does your child make errors in pronouns or articles (he/she)? |  |
| **Attention** | |
| **Please rate your child on a scale of 0-10, with decimals allowed, based on the following:** | Score |
| 1. Is it difficult for your child to pay attention to details, or do they make careless mistakes in homework? |  |
| 2. Is it challenging for your child to maintain focus on tasks or games? |  |
| 3. Does your child have difficulty listening when others speak directly to them? |  |
| 4. Does your child struggle with completing homework? |  |
| 5. Is it hard for your child to organize tasks or activities, or start several things without finishing any? |  |
| 6. Does your child avoid or unwillingly complete tasks that require sustained mental effort (such as homework)? |  |
| 7. Does your child lose or forget things related to daily activities (swimsuit for the pool, class assignments, pen/notebook, etc.)? |  |
| 8. Does your child want to give answers before questions are completed? |  |
| 9. Is it challenging for your child to wait their turn or sit calmly in a waiting room? |  |
| 10. Does your child interrupt others or impose their presence (e.g., interrupting conversations or games)? |  |
| 11. Does your child challenge what adults or authority figures say, violate their rules, or argue/disobey? |  |
| 12. Does your child intentionally annoy others? |  |
| 13. Does your child blame others for their mistakes or rudeness? |  |
| 14. Is your child sensitive, easily angered, or provoked by others? |  |
| 15. Does your child make sounds with their mouth, hands, or any other part of their body? |  |
| 16. Is your child uncooperative? |  |
| 17. Is your child restless, impulsive, or inattentive? |  |
| 18. Does your child tease, disturb, or disrupt other children or their activities? |  |
| 19. Does your child have a poor sense of time (e.g., not knowing if 5 minutes or 20 minutes have passed)? |  |
| 20. Does your child postpone tasks they don't like? |  |
| **Coordination** | |
| **Please rate your child on a scale of 0-10, with decimals allowed, based on the following:** | Score |
| 1. Is it difficult for your child to throw a ball with good control and accuracy? |  |
| 2. Is it hard for your child to catch a small ball (e.g., a tennis ball) from a distance of 2 to 3 meters? |  |
| 3. Does your child find it challenging to accurately hit a nearby ball or shuttlecock with a bat or racket? |  |
| 4. Is it difficult for your child to navigate obstacles found in a garden or playground? |  |
| 5. Does your child struggle to run as quickly as other same-age children of the same gender? |  |
| 6. Is your child challenged in planning movements or coordinating their body to execute such plans, making it hard to complete tasks effectively (e.g., building a house with blocks on a frame)? |  |
| 7. In class, does your child find it difficult to keep up with other children in quick printing, writing, or drawing? |  |
| 8. Does your child find it hard to print or write clear, accurate, and precise letters, numbers, and words? |  |
| 9. Does your child have difficulty coordinating coloring and drawing, producing recognizable figures? |  |
| 10. When printing, writing, or drawing, is it challenging for your child to apply appropriate effort and tension? |  |
| 11. Does your child find it hard to cut pictures and shapes accurately? |  |
| 12. Does your child have difficulty participating in sports or activities that require good motor skills? |  |
| 13. Does your child need more practice or time than other children to acquire new motor skills at the same level? |  |
| 14. Does your child struggle to organize, put on shoes, or get dressed quickly? |  |
| 15. Is your child described as a "bull in a china shop" (clumsy and awkward)? |  |
| 16. Does your child become fatigued easily, look lazy, or fall off chairs if they need to sit for an extended period? |  |

**Supplementary Table 4 Parent Ten Program Self-Assessment Form**

| **Evaluation Categories:** | **Score** | **Specifications** |
| --- | --- | --- |
| 1. Gross Motor and Fine Motor Skills |  |  |
| 2. Energy Level |  |  |
| 3. Alertness (Responsiveness and Reaction Capability) |  |  |
| 4. Speech and Pronunciation |  |  |
| 5. Language Comprehension |  |  |
| 6. Psychological State(Add points for positivity (e.g., sunshine), deduct for anxiety.) |  |  |
| 7. Emotional Temperament |  |  |
| 8. Social Skills |  |  |
| 9. Attention |  |  |
| 10. Learning Ability |  |  |
| 11. Other Aspects |  |  |
| Total Score: |  |  |
| The sum of scores from the 11 categories.  For each category, a higher score indicates better performance or ability, while a lower score indicates challenges or difficulties. Fill in each category's score and provide details for "Other Aspects" if needed. | | |

Scoring System:

- Rate each of the 11 items on a scale from 0 to 10, with 10 being the highest score.
- Full marks are 10 points for each item.
- Deduct points for weaknesses or difficulties, and add points for strengths or positive aspects.
- Provide additional details or comments for the "Other Aspects" category.

**Supplementary Table 5 Participant Behavioral Indicator Scores Before and After Cross-sectional Study Training**

**Supplementary Table 5** Scores of behavioral indicators of participants in the experimental group.

| **No.** | **ABC** | | **CARS** | | **Frequency band** | | | | | | | |
| --- | --- | --- | --- | --- | --- | --- | --- | --- | --- | --- | --- | --- |
|  |  |  |  |  | **LF** | | **MF** | | **HF** | | **LHT** | |
|  | Pre | Post | Pre | Post | Pre | Post | Pre | Post | Pre | Post | Pre | Post |
| Ⅰ | 83.0 | 71.0 | 38.0 | 31.5 | 3.0 | 4.0 | 3.2 | 5.9 | 3.0 | 4.3 | 3.0 | 5.6 |
| Ⅱ | 69.0 | 47.0 | 31.0 | 24.5 | 7.0 | 6.0 | 6.0 | 7.8 | 6.0 | 7.3 | 6.0 | 7.8 |
| Ⅲ | 112.0 | 91.0 | 38.5 | 35.0 | 7.0 | 8.0 | 5.8 | 8.7 | 6.0 | 7.7 | 5.8 | 8.6 |
| Ⅳ | 134.0 | 123.0 | 36.0 | 32.0 | 5.0 | 5.0 | 5.8 | 6.9 | 2.4 | 2.7 | 3.6 | 4.8 |
| Ⅴ | 87.0 | 54.0 | 34.0 | 29.0 | 4.0 | 7.0 | 4.2 | 8.0 | 4.3 | 7.7 | 4.2 | 8.0 |
| Ⅵ | 129.0 | 111.0 | 40.5 | 39.0 | 4.0 | 5.0 | 3.8 | 5.5 | 3.1 | 4.5 | 3.0 | 5.0 |
| Ⅶ | 129.0 | 118.0 | 43.0 | 38.0 | 7.0 | 7.0 | 6.0 | 7.0 | 5.7 | 5.5 | 6.2 | 6.0 |
| Ⅷ | 125.0 | 86.0 | 35.0 | 32.0 | 8.0 | 8.0 | 7.3 | 7.7 | 6.3 | 6.8 | 6.7 | 7.2 |
| Ⅸ | 69.0 | 42.0 | 32.0 | 29.0 | 7.0 | 8.0 | 4.6 | 8.0 | 4.7 | 8.7 | 4.7 | 8.2 |
| Ⅹ | 69.0 | 51.0 | 30.0 | 27.5 | 7.0 | 7.0 | 8.6 | 9.3 | 8.1 | 7.8 | 8.0 | 8.5 |
| Ⅺ | 88.0 | 61.0 | 35.0 | 31.0 | 8.0 | 8.0 | 5.7 | 6.5 | 5.3 | 7.7 | 5.0 | 7.0 |
| Ⅻ | 152.0 | 127.0 | 47.0 | 37.0 | 8.0 | 8.0 | 4.7 | 6.4 | 4.7 | 6.0 | 4.5 | 6.0 |
| XIII | 72.0 | 51.0 | 31.0 | 21.0 | 6.0 | 6.0 | 8.0 | 8.4 | 5.3 | 6.3 | 6.8 | 7.5 |
| XIV | 116.0 | 76.0 | 37.0 | 30.0 | 6.0 | 6.0 | 4.8 | 6.7 | 1.3 | 4.0 | 3.2 | 5.5 |
| XV | 153.0 | 123.0 | 39.5 | 35.5 | 3.0 | 5.0 | 4.2 | 6.5 | 3.3 | 4.7 | 3.8 | 5.8 |

*LF, low-frequency band; MF, mid-to-high frequency band; HF, high-Frequency band; LHT, lateralization, and hearing thresholds (Same as below).*

**Supplementary Table 6 Longitudinal study pre- and post-training participant autism scores**

**Supplementary Table 6** Longitudinal participants were scored on the odd-period parent ten-item rating scale.

| No. | Training Session | Session 1 | Session 3 | Session 5 | Session 7 | Session 9 | Session 11 |
| --- | --- | --- | --- | --- | --- | --- | --- |
| I | 7 | — | — | — | — | — | — |
| II | 8 | — | — | — | — | — | — |
| III | 7 | — | — | — | — | — | — |
| IV | 6 | — | — | — | — | — | — |
| V | 10 | 36.0 | — | 44.0 | 48.0 | 56.0 | — |
| VI | 10 | 47.0 | 48.0 | 53.0 | 55.5 | 63.0 | — |
| VII | 8 | 55.0 | 59.0 | 66.0 | 68.0 | 73.0 | — |
| VIII | 8 | 50.0 | 62.0 | 68.0 | 72.0 | — | — |
| IX | 13 | 33.0 | 52.0 | 58.0 | 59.5 | 59.0 | 59.0 |
| X | 12 | 55.0 | 68.0 | 71.0 | 78.0 | 78.0 | 80.0 |
| XI | 6 | 50.0 | 54.5 | 60.0 | — | — | — |
| XII | 7 | 76.0 | — | 76.0 | 75.0 | — | — |
| XIII | 8 | 41.0 | 42.0 | 45.0 | 50.0 | — | — |
| XIV | 8 | 76.0 | 74.8 | 74.3 | 70.6 | — | — |
| XV | 7 | 62.5 | 70.0 | 79.0 | 79.0 | — | — |
| XVI | 8 | 56.0 | 58.0 | 63.0 | 61.0 | — | — |
| XVII | 8 | 65.0 | 69.0 | 69.0 | 72.0 | — | — |
| XVIII | 6 | — | — | — | — | — | — |
| XIX | 6 | — | — | — | — | — | — |
| XX | 8 | 58.0 | 54.0 | 58.0 | 58.0 | — | — |
| XXI | 6 | — | — | — | — | — | — |
| XXII | 8 | — | 68.0 | 59.0 | 59.0 | — | — |
| XXIII | 8 | 55.0 | — | 64.0 | 62.0 | — | — |
| XXIV | 6 | — | 72.0 | 69.0 | — | — | — |
| XXV | 6 | — | 48.0 | 59.0 | — | — | — |

**Supplementary Table 7 Longitudinal Study Pre-Training Participant TOMATIS Test Behavioral Response Record and Basis Situation**

To protect the privacy of the subjects, code words are used hereafter.

I: Poor in gross motor skills, normal in fine motor skills. Family history of tic disorders, and occasional tic-like behaviors. Non-verbal; dislikes wearing headphones, and reacts normally to sounds but doesn't like wearing headphones.

II: Behavior is relatively stable. Cognitive abilities: unable to understand others' speech intonation, strange speech expressions, lack of concentration, poor cognitive abilities. In auditory testing, can wear headphones but cannot respond appropriately.

III: Behavior is chaotic and easily stimulated. Cognitive abilities: low arousal, poor memory, basically non-verbal, poor understanding, imbalance between left and right brain. In testing, can wear headphones but struggles to respond.

IV: Has strabismus, purposeless movements, poor proprioception, and vestibular sense. No interest in books but likes children's songs. Cognitive: sensitive to sound. In testing, uncomfortable with headphones, and needs guidance to respond to auditory stimuli.

V: Easily stimulated, fearful of new environments, exhibits avoidance and fearful sounds, pulls his hair, poor sleep, malnutrition, and problems in both gross and fine motor skills. Cognitive: sensory integration issues, non-verbal but occasionally understands mother's meaning. Dislikes headphones, cannot maintain a stable state, and struggles to respond even with guidance.

VI: Behavior: attention and interaction with people are weak, temperamental, and impulsive. In testing, accepts headphones, and answers questions without prompting.

VII: Poor attention, narrow interests, soft and vague movements. Accepts headphones and, after calming down, can answer which ear heard the sound.

VIII: Behavior: needs assistance with eating and sleeping, easily agitated, sometimes aggressive. Cognitive: fluent speech but with strange expressions. In testing, can wear headphones and answer questions about what was heard.

IX: Non-verbal, lacks understanding of others' intentions, gaze does not focus on others. In testing, can wear headphones but struggles to respond clearly.

X: Behavior: poor sleep, wakes up early 1-2 times a month. Cognitive: delayed language development, almost non-verbal. In testing, can wear headphones and answer questions with guidance.

XI: Behaviorally, he cannot communicate effectively, has poor eye gaze, has difficulty adapting to daily life, needs to be taken care of by others, and occasionally wets his pants; cognitively, he has poor verbal comprehension, cannot communicate others' intentions, and is immersed in his own world; when tested for response, he was able to accept wearing a headset, but was unable to answer the questions, and could not answer the questions guided by the headset when it was put on.

XII: Behaviorally, verbal fluency, but not active, emotionally unstable, easily rushed; uncomfortable with headphones on when testing responses, occasionally able to answer questions when guided with headphones on.

XIII: Behaviorally, unable to sit comfortably and needs to be attended to by others; cognitively, unable to communicate effectively and has cognitive difficulties; when tested for response, unable to answer questions clearly without accepting to wear headphones.

XIV: Behaviorally, normal eye gaze, weak gross motor development, slightly slower reflexes; cognitively, able to understand most conversations, occasional anxiety, easily distracted, slower to learn; when tested for reflexes, able to accept wearing headphones, able to answer questions about what is being heard with guidance.

XV: Behaviorally, sleep is not steady. Cognitively, visual processing is slow, sensory integration disorder, verbal fluency, but attention is not focused, able to understand most of the meaning of others' words; when tested for response, able to accept wearing headphones, able to answer what he hears with headphones on.

XVI: Behaviorally, he talks less, has a history of respiratory infections complicated by otitis media and allergic rhinitis, deals with people in an inappropriate way, loves to push things, and has more small movements; when tested for response, he was able to accept wearing headphones and was able to respond to what he heard when wearing headphones.

XVII: Behaviorally, he has had poor sleep since childhood, frequent bedwetting and constipation, poor fine motor development, slow threading of beads, inflexible grip on spoons, and better jumping rope; cognitively, he has sensorimotor incoordination, vestibular delays, inability to understand others' words well, slower reaction time, insufficient social cognition, and myelin dysplasia as shown by MRI. When tested for response, able to accept headphones, able to answer some questions in small amounts under guidance, but not able to express clearly.

XVIII: behaviorally, presents lazy and weak state, vague speech articulation, more stable emotion; cognitively, has difficulties in math learning and language comprehension, inattentive, poor alertness, greater MRI shows myelin dysplasia, delayed brain development, localized brain white matter developmental anomalies, abnormal ear response, poor concentration; when tested for response, is able to accept wearing earphones, describes which side of the ears when hearing sounds, answers were unclear.

XIX: Behaviorally, able to carry on a conversation, not attentive to others; Cognitively, inattentive, not actively talking; When tested for response, able to wear headphones, able to answer most of the questions when wearing headphones.

XX: Behaviorally, unable to stand, weak self-control, unable to respond to others' words; cognitively, basically no verbal expression, poor attention span, presence of sound sensitivity, abnormal reaction to the sound of flushing the toilet; when tested for response, able to accept wearing earphones, but has difficulty answering questions under guidance, and answered questions incorrectly.

XXI: Behaviorally, hyperactive, unable to be quiet, gaze cannot be directed, eyes generally look at floor tile seams; cognitively, lack of concentration, susceptible to visual stimuli, easily collapses in unfamiliar environments; when tested for response, able to accept headphones for auditory testing, after calming down, gradually stabilized, can answer which ear heard the sound.

XXII: Behaviorally, his gross motor development is not as good as that of his peers, he is inattentive, slow in doing things, his voice tone is normal, but his final word trails off; cognitively, he is able to understand most of what others say, there is a gap between his interests and those of his peers, and he does not integrate into the group well. When tested for response, able to wear headphones, able to actively answer what they hear when wearing headphones.

XXIII: Behaviorally, hyperactive, less developed than his peers in gross motor skills, poor chewing ability, stiff facial movements, sings, interested in piano, enjoys music, indulges in his favorite things; cognitively, can identify numbers and shapes, can communicate in general speech but does not initiate, poor concentration, MRI shows myelin dysplasia; when tested for response, can accept wearing headphones, can answer questions with guidance, can accept wearing headphones. Can answer questions when guided. Accepts sounds played on headphones;

XXIV: Behaviorally, very hyperactive, needs parents' attention all the time, basically non-verbal, does not respond to others, emotionally positive on issues of interest; cognitively, less negative emotions, inattentive, cognitively difficult on logical issues that require thinking; when tested for response, puts on earphones for a period of time and then removes them by himself, not capable of answering questions.

XXV: Behaviorally, he cannot speak, his speech is unclear, he has difficulty adapting to life, and he sleeps poorly. From time to time, he has a socially oriented smile and reflexive behavior after partial stimulation. Cognitively, he is not able to distinguish between himself and others, easily afraid, inattentive, occasionally has poor emotional state, has no interest in peers, and has weak learning ability. When tested for response, able to wear headphones, unable to remove headphones autonomously, unable to cooperate in answering questions while wearing headphones.
